# Mitochondria-targeted strategies in tumor immunity

**DOI:** 10.3389/fimmu.2025.1646138

**Published:** 2025-10-30

**Authors:** Xudong Cheng, Yian Wang, Bryon Johnson, Ming You

**Affiliations:** ^1^ Department of Pharmacy, Suzhou Traditional Chinese Medicine (TCM) Hospital Affiliated to Nanjing University of Chinese Medicine, Suzhou, China; ^2^ Center for Cancer Prevention, Houston Methodist Neal Cancer Center, Houston Methodist Hospital, Weill Cornell Medicine, Houston, TX, United States; ^3^ Division of Hematology and Oncology, Department of Medicine, Medical College of Wisconsin, Milwaukee, WI, United States

**Keywords:** mitochondria targeted, triphenylphosphonium, TME, cancer, immunotherapy

## Abstract

Mitochondria, as regulators of cellular energy production and metabolism, play a crucial role in tumor growth and survival. Tumors are reprogrammed to accommodate rapid proliferation through the Warburg effect. This reprogramming leads to the accumulation of metabolites such as lactate and ketone bodies, thereby lowering the pH of the tumor microenvironment, inhibiting the activity of effector T cells and NK cells, while promoting the infiltration of regulatory T cells and MDSCs, forming an immunosuppressive microenvironment. ROS produced by mitochondria can affect immune cell function by modulating their signaling pathways. Mitochondria also release DAMPs, which activate the antigen-presenting capacity of dendritic cells and initiate anti-tumor immune responses. Currently, various methods have been employed, such as DLCs modifications and mitochondrial targeted delivery, which enable drugs to penetrate the lipid bilayer and enter the mitochondria, thereby helping to reduce immunosuppression in the tumor microenvironment. In this review, we will discuss the impact of mitochondria on tumor immunity, strategies to target tumor cell mitochondria, and progress on the discovery of mitochondria-targeted drugs to enhance tumor immunity, providing potential directions for developing new cancer therapeutic strategies.

## 1 Introduction

Mitochondria are essential intracellular organelles that primarily facilitate energy production and metabolic regulation (1). They generate adenosine triphosphate (ATP) via oxidative phosphorylation (OXPHOS), thereby providing cells with the energy required for various functions (2). Moreover, mitochondria modulate diverse physiological processes including calcium homeostasis, redox balance, apoptosis and immune responses through mitochondrial DNA (mtDNA), reactive oxygen species (ROS), and metabolite signaling pathways (3, 4). Consequently, abnormal mitochondrial function may contribute to the pathogenesis of various diseases, including cancer.

Mitochondria play a particularly critical role in tumorigenesis and progression (5). Tumor cells frequently undergo metabolic reprogramming to support rapid proliferation, exemplified by the “Warburg effect,” wherein cells preferentially utilize glycolysis over OXPHOS despite adequate oxygen availability (6). This metabolic shift not only sustains tumor cell growth and survival but also modulates immune cell function within the tumor microenvironment (TME) (7). For instance, hypoxia in the TME can induce T-cell exhaustion, thereby impairing anti-tumor immune responses (8). Additionally, mitochondrial dysfunction is closely associated with immune escape mechanisms that promote tumor progression and metastasis (9).

Recent years have witnessed significant advancements in tumor immunotherapy, particularly with the advent of immune checkpoint inhibitors. Nevertheless, many patients exhibit either primary or acquired resistance to such therapies, a phenomenon closely linked to the immunosuppressive nature of the TME (10). Immune cells, such as tumor-associated macrophages (TAMs), rely on mitochondrial metabolic functions to maintain their immunosuppressive activity (11). Therefore, targeted therapies aimed at modulating mitochondrial function may overcome immune resistance by reprogramming immunosuppressive cells (12). For example, recent progress in mitochondrial-targeted metabolic reprogramming has demonstrated that enhancing T-cell bioenergetics can restore antitumor activity (13). Furthermore, mitochondrial-derived ROS modulates immune cell function via redox signaling; low ROS levels promote T-cell exhaustion, whereas normal ROS levels enhance antigen presentation by dendritic cells (DCs) (14). Given the critical role of mitochondria in tumor metabolism and immunotherapy, elucidating how mitochondria-targeting strategies influence tumor immunotherapy represents a promising area of research. In this review, we will explore the emerging role of mitochondria in tumor immunotherapy and discuss the recent advances in mitochondria-targeted drugs that enhance tumor immunity, thereby providing important directions for future therapeutic strategies.

## 2 Mitochondria and tumor immunity

**Figure 1:**
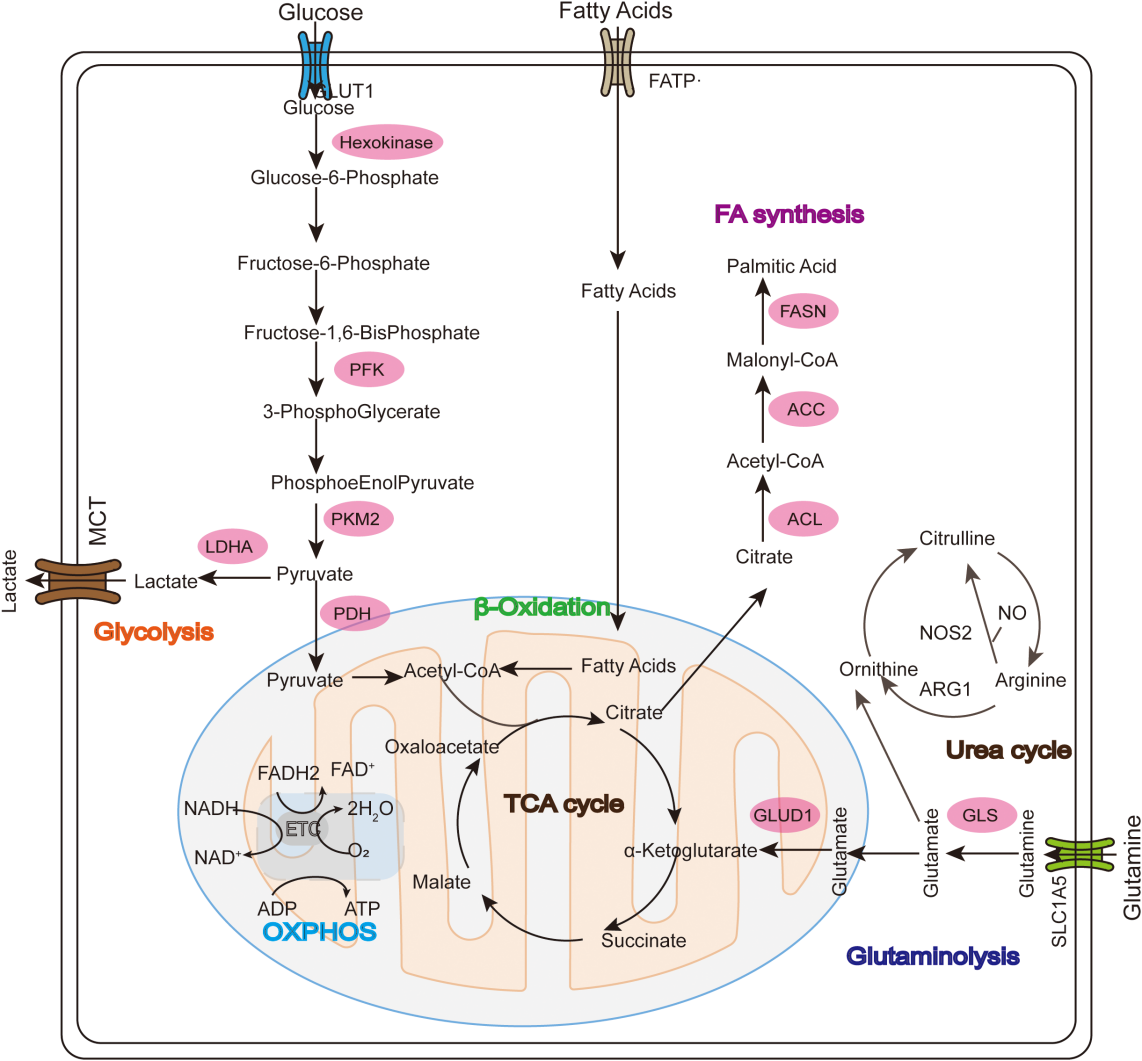
Glucose, fatty acid, and glutamine metabolism in mitochondria during tumor development. In the Warburg effect, unlike normal differentiated cells, glucose enters the cell through GLUT1 and mainly relies on mitochondrial oxidative phosphorylation to provide energy for the cell, while most tumor cells rely on aerobic glycolysis and are eventually oxidized to lactate instead of acetyl-CoA (ac-COA). The main substrate for lipid synthesis is cytoplasmic acetyl-CoA synthesized through a series of reactions. Fatty acid oxidation (FAO) allows long-chain FA to be converted into acetyl-CoA in the mitochondria and enter the TCA cycle to generate ATP and malic enzyme-dependent NADPH. Glutamate is then converted into α-ketoglutarate (α-KG) through two different pathways and can participate in the TCA cycle as a replenishing substrate. Glutamine sequentially catalyzes the formation of arginine (Arg) through citrulline (Cit), and then continues to decompose under the action of arginase (ARG) to produce urea and ornithine (Orn), thus forming the urea cycle.

Tumor cells adapt to increasing energy and biosynthetic demands by reprogramming relevant metabolic pathways (15). Nutrient depletion and overproduction of metabolic byproducts driven by tumor development in the TME help to establish an immunosuppressive TME by regulating the metabolic reprogramming of tumor-infiltrating immune cells and associated signaling activation to control the polarization of different types of immune cells, ultimately resulting in metabolic derangement-mediated deficiencies and decreased anti-tumor immune responses (16). Mitochondria, as intracellular organelles with diverse biological functions and highly variable, have key regulatory roles in metabolism and activating immune cells (17). Glucose, fatty acid and amino acid metabolism are abnormal during tumor development and progression (18) (**Figure 1**). ROS-induced mtDNA damage impairs mitochondrial OXPHOS, forcing tumor cells to rely on glycolysis for ATP production (19). Abnormal mitochondrial function in the TME is an important cause of cancer formation, progression and metastasis.

### 2.1 Mitochondrial metabolic regulation of immune cells

**Figure 2:**
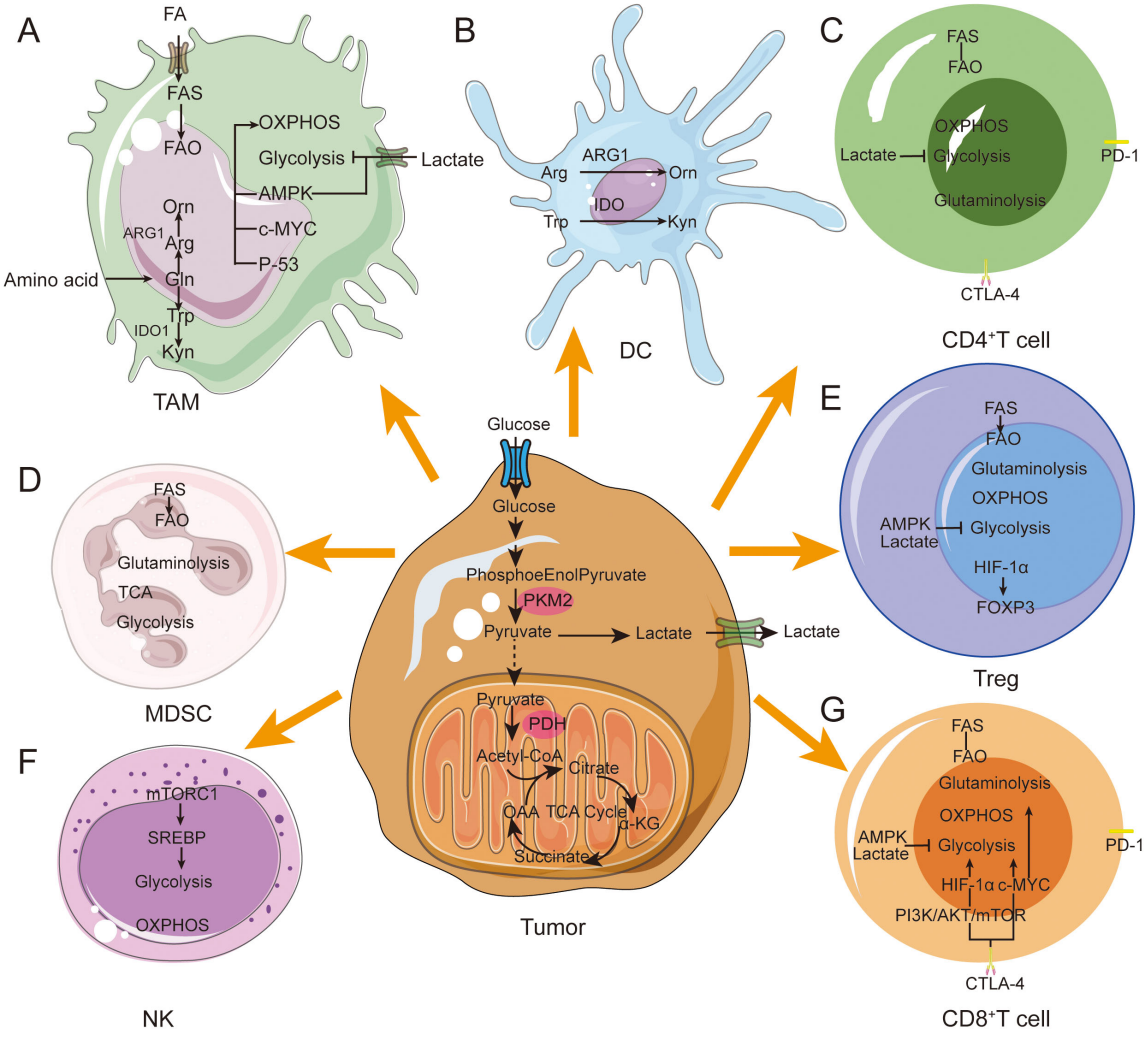
Metabolic reprogramming in immune cells during tumor progression. Immune cells in the TME achieve immunosuppressive and pro-tumor phenotypes through metabolic reprogramming. **(A)** M1 macrophages prefer glycolysis and secrete a large amount of lactate. M2 macrophages tend to show enhanced fatty acid oxidative phosphorylation ability. M2 macrophages mainly rely on FAO, OXPHOS and glutamine metabolism. **(B)** Immediately after DC activation, glycolysis increases rapidly to provide ATP. DCs express ARG1 and IDO enzymes. Hydrolyze arginine and tryptophan. **(C)** High lactate concentration blocks CD4+ T cell glycolysis. CD4+ T cells increase lipid uptake leading to a metabolic shift toward FAO. **(D)** MDSCs express ARG1 and IDO enzymes. Hydrolyze arginine and tryptophan. Tumor-infiltrating MDSCs exhibit enhanced glycolysis and OXPHOS. **(E)** In Tregs, FOXP3 expression inhibits glycolysis while promoting OXPHOS. FASN overexpression enhances lipid metabolism. **(F)** NK-mediated glycolysis via mTORC1 signaling. and OXPHOS. **(G)** High lactate concentrations block T cell glycolysis. Activated CD8+ T cells convert glucose and glutamine into biomass and rely on the Pl3K and AKT pathways. CD8+ T cells tend to FAO increase lipid uptake.

T cells rely on OXPHOS and fatty acid oxidation in the resting state, but switch to aerobic glycolysis and fatty acid synthesis upon activation to support proliferation (20, 21) (**Figure 2**). During T cell activation, mitochondria accumulate in the immune synapse formed by T cells and antigen-presenting cells (APCs), and activation of the T cell receptor stimulates an increase in mitochondrial fission, which increases the number of mitochondria and cristae loosening, and generation of ROS and ATP, which are essential in maintaining calcium homeostasis and regulating its downstream-related signaling (22). During the transformation of CD8^+^ T cells from effector T cells to memory T cells, activation of Sirt3, a mitochondrial deacetylase, reduces protein acetylation, which enhances OXPHOS activity and generation and survival of memory T cells, resulting in increased anti-tumor immune activity (23). In contrast, competition of tumor cells for glucose and other nutrients in the TME suppresses the metabolism and function of immune cells (24). Hypoxia in the TME promotes mitochondrial structural damage and reduces ATP production by down-regulating MYC expression levels, which induces T-cell exhaustion (TExh) and anti-tumor dysfunction of CD8^+^ T cells. Tumor-infiltrating T cells are in a state of high oxidative stress for long periods of time due to glucose and oxygen-deficient environment-mediated metabolic insufficiency and impairment of mitochondrial function and quality (25). In addition, peroxisome proliferator-activated receptor γ coactivator-1α (PGC-1α), a key regulator of mitochondrial biogenesis, is upregulated in CD8^+^ T cells in the TME resulting in their dysfunction. This dysfunction can be reversed/rescued by enhancing cellular expression of PGC1α, which increases CD8^+^ T cell anti-tumor activity (26).

Natural killer (NK) cells are cytotoxic lymphocytes, and their cellular activity is significantly correlated with levels of glucose metabolism. When glucose levels are elevated, NK cell activity is significantly enhanced. After activation of NK cells, intracellular sterol regulatory element binding protein (SREBP) binds to and upregulates its mechanistic target rapamycin complex 1 (mTORC1) expression and enhances aerobic glycolysis and OXPHOS metabolism (27). The transcription factor cMyc can significantly increase NK cell metabolism; if the c-Myc protein is defective, NK cells will reduce their expression of key gluconeogenesis and mitochondrial enzymes, leading to impaired immune function (28). When NK cells transition to the memory stage, mitochondrial autophagy-related proteins Bnip3-Bnip3L promote their transition by inducing mitochondrial autophagy to remove damaged mitochondria and reduce the generation of ROS (29). Studies have shown that in a hypoxic TME, the mitochondrial morphology of tumor-infiltrating NK cells shows significant fragmentation and division compared to normal NK cells. These changes in mitochondrial morphology significantly reduce the ability of NK cells to mediate tumor immune surveillance (30).

Mitochondria also play a key role in macrophage polarization. In the early stage of tumor formation, pro-inflammatory cytokines such as toll-like receptor (TLR) agonists can promote the polarization of TAM to an M1 phenotype, and nitric oxide (NO) and ROS produced by M1 type macrophages can significantly inhibit the proliferation and induce tumor cell death (31). During tumor progression, interleukin (IL)-4 and colony stimulating factor 1 (CSF1) induce the polarization of TAM to M2 phenotype. M2 macrophages secrete epidermal growth factor (EGF), matrixmetalloprotein9 (MMP-9), and other proteins to inhibit anti-tumor immunity and promote tumor progression (32). M2 macrophages rely on the OXPHOS metabolic pathway for energy supply (33), which is linked to fatty acid oxidation (FAO) and characterized by high expression of CD36. CD36 promotes the mitochondrial OXPHOS process, resulting in mitochondrial fusion and lengthening (34). Additionally, M2 macrophages synthesize large amounts of arginase (ARG) and indoleamine2,3-dioxygenase1 (IDO1), which deplete arginine and tryptophan respectively, leading to immune dysfunction (35). FAO plays a key role in human M2 macrophage function by enhancing IL-1β secretion to promote cancer cell migration (36).

The metabolic shift from OXPHOS to glycolysis and dynamic changes in mitochondrial morphology lead to alterations in immune cell polarity and phenotype, which in turn affects the biology of immune cells. Therefore, studying the role of mitochondrial metabolism is important for understanding regulation of tumor immunity and developing new drugs that can promote tumor immunity.

### 2.2 Mitochondrial ROS in tumor immunity

Mitochondria are the main intracellular ROS-generating organelles, producing ROS through the electron transport chain (ETC) and OXPHOS during aerobic respiration (37). ROS have dual roles in tumorigenesis and progression. Low levels of ROS act as important cellular signaling molecules involved in multiple life activities such as gene expression, cell proliferation, differentiation, and stress responses. However, when the intracellular levels of ROS are too high, oxidative damage to nucleoplasm, mitochondrial DNA, proteins and lipids occurs, which ultimately leads to cellular damage. High levels of ROS facilitate tumorigenesis by promoting tumor cell proliferation, migration, invasion and angiogenesis, inflammatory responses and immune escape, helping tumor cells adapt to the harsh TME. In addition, ROS-mediated inflammatory responses can also change the composition of immune cells in the TME and enhance immune suppression (38). Therefore, maintaining a balance between intracellular ROS production and consumption is essential for maintaining cellular homeostasis and organismal health.

#### 2.2.1 ROS and formation of a tumor immunosuppressive microenvironment

As highlighted in the previous section, ROS plays a central regulatory role in the TME and drives cancer development and progression (39). Tumor cells adapt to the high reactive oxygen environment and avoid cell death by inducing the secretion of inflammatory cytokines, stabilizing hypoxia-inducible factor-1α (HIF-1α), activating AMP-activated protein kinase (AMPK) signaling, and promoting the production of nicotinamide adenine dinucleotide phosphate (NADPH), which in turn promotes tumor metastasis and angiogenesis (40). In addition, ROS regulates the activation status of immune cells in the TME that affect cancer progression. High ROS levels oxidize major histocompatibility complex (MHC) class I molecules, which impairs antigen peptide loading and T-cell receptor(TCR)-MHC/peptide complex stability (41). Tumor cells and immunosuppressive cells in the microenvironment act synergistically to induce mitochondrial ROS (mtROS) generation, aiding in the establishment of immune tolerance (42). Lon protease in the mitochondrial quality control system induces ROS generation by interacting with multiple proteins, mediating activation of the NF-κB signaling axis and enhancing downstream signaling activity to promote tumorigenesis (43). Highly expressed HIF-1α promotes mtROS production by inducing Lon protease expression (44). Lon protease binds PYCR1, a key enzyme in proline metabolism, enhancing NADPH consumption and promoting electron leakage in the ETC, thereby elevating mtROS (45).

#### 2.2.2 Effects of mtROS on immune cell activation in the TME

**Figure 3:**
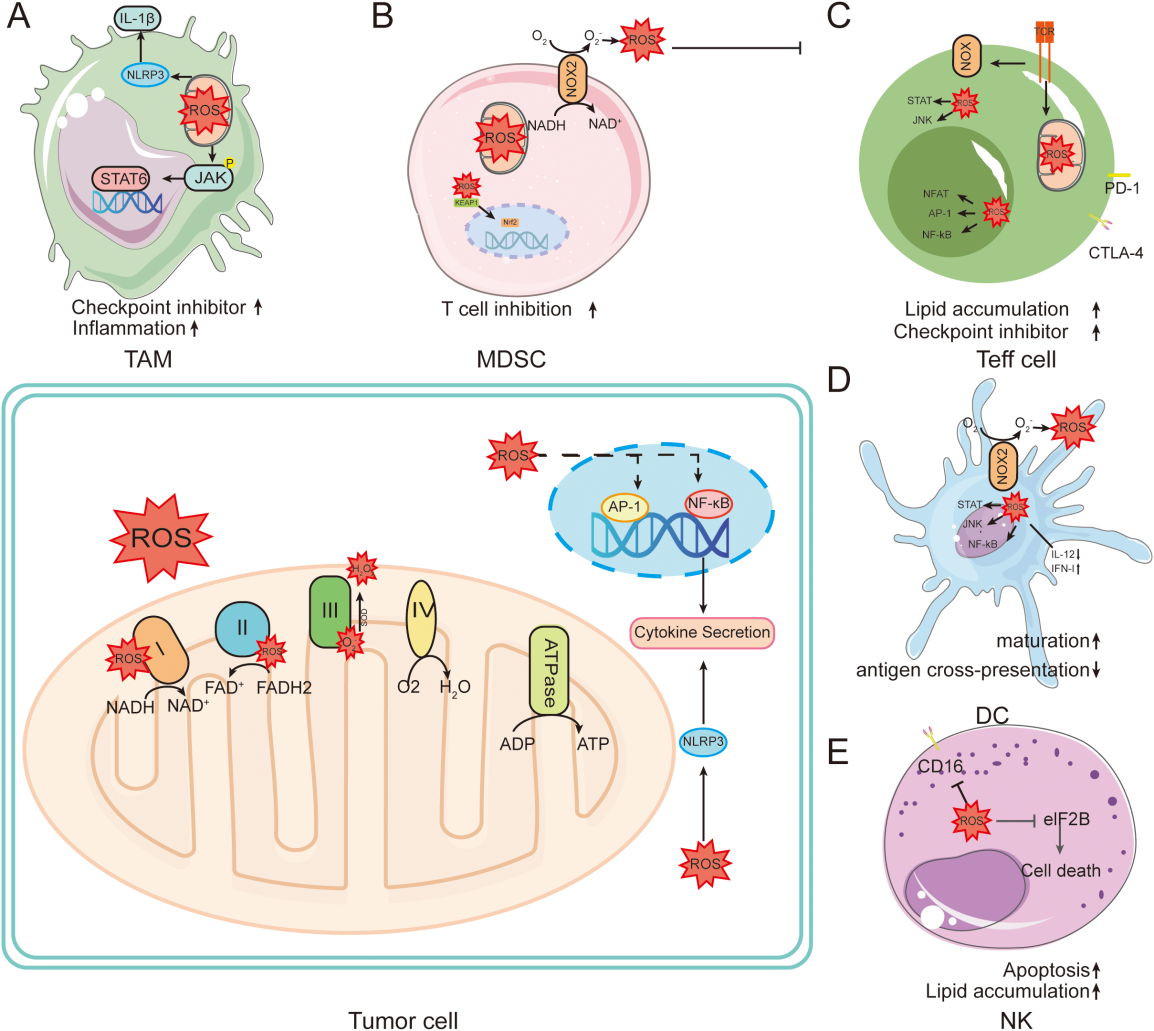
Role of ROS in tumors and immune cells. ROS are mainly generated in the electron transport chain on the inner membrane of mitochondria during oxidative phosphorylation. This leads to intracellular oxidative stress. **(A)** In macrophages, ROS induce powerful intracellular changes by activating signaling pathways such as NF-kB, AP-1, and NRF2. Activation of NF-kB and AP-1 leads to the production of key pro-inflammatory cytokines. **(B)** NOX family members in MDSC cells directly mediate the production of ROS, further promoting the inflammatory cascade. **(C)** TCR engagement can mediate the production of ROS through NOX family members, increasing the activity of NFAT, Myc, and mTOR. **(D)** ROS can affect DC maturation, antigen presentation, NF-κB activation, and induction of anti-inflammatory cytokines. **(E)** ROS induced in NK promotes NK cell apoptosis.

To avoid the deleterious effects of high ROS levels on immune cells, there exists a set of strict regulatory mechanisms in the organism to maintain a delicate balance between immune cell activity and ROS levels (46). Precise control of ROS levels in NK cells and T lymphocytes prevents their damage to other lymphocytes (**Figure 3**). In the tumor microenvironment, IL-15 has been shown to induce NK cells to enhance their resistance to oxidative stress and protect against ROS via the thioredoxin system (47). During anti-tumor immunity, activated T lymphocytes and NK cells recruit neutrophils and macrophages by increasing ROS production, ultimately killing tumor cells (48). On the other hand, elevated ROS inhibits prolyl hydroxylases (PHDs), stabilizing HIF-1α to drive myeloid-derived suppressor cell (MDSC) differentiation (49). For example, tumor-associated fibroblasts promote the transformation of peripheral monocytes into MDSCs by increasing their oxidative stress, thereby inhibiting the proliferation of CD8^+^ T cells and promoting tumor progression (50).

ROS plays an important regulatory role in T cell activation, promotion of T cell antigen-specific proliferation and apoptosis (51). Moderate levels of ROS are essential for the normal activation and differentiation of T lymphocytes, whereas high levels of ROS promote T cell apoptosis by up-regulating the apoptosis-related factor Fas and down-regulating expression of the anti-apoptotic protein Bcl-2 (52). In addition, extracellular ROS affects T cell activation by altering the immunogenicity of antigenic peptides in APCs (53). During immunogenic cell death (ICD), intracellular damage-associated molecular patterns (DAMPs) such as ATP, endoplasmic reticulum calmodulin, and high mobility group protein B1 leaks to the extracellular space, which in turn activates DCs by interacting with its receptors and triggers anti-tumor immune responses in T lymphocytes (54). Nanoparticle-delivered catalase scavenges extracellular H_2_O_2_, enhancing T cell infiltration and reversing immunosuppression (55). In addition, reduced glutathione deficiency in regulatory T cells (Treg) leads to abnormal serine metabolism and down-regulates transcription factor forkhead box P3 (Foxp3), which ultimately attenuates the immunosuppressive function of Treg (56). These studies suggest that ROS levels and sustained generation capacity have a key role in ICD.

ROS have long been considered to be harmful metabolites of mitochondria, but recent studies have shown that mtROS have a necessary signaling role in preventing excessive immune responses, and in particular ROS play a key role in regulating macrophage immune responses (57). Under normal conditions, ROS affects macrophage polarization by modulating relevant signaling pathways (58). ROS also have important regulatory roles in macrophages subsets. For example, M1-type macrophages generate ROS through NADPH-oxidase (NOX) 2 signaling, which activates NF-κB signaling and enhances cellular phagocytosis (59). In contrast, high levels of ROS have harmful effects on macrophages (60). During tumorigenesis, macrophages become an important immune cell population for maintaining immune homeostasis in the TME. Tumor cells remodel the peripheral and distal TME by secreting tumor-derived factors, which stimulate the activation of both monocytes and macrophages in the microenvironment and accelerate tumor progression (61). Although TAM can exhibit both pro-inflammatory M1-type and anti-inflammatory M2-type polarized forms, it is generally accepted that TAM exhibit similar functions to M2-type macrophages, promoting tumor growth, metastasis, angiogenesis, and immunosuppression by secreting cytokines, chemokines, and proteases (62). Mitochondrial Lon protease is upregulated in M2-type macrophages, suggesting that during tumorigenesis macrophages may regulate Lon expression through multiple signals, inducing mtROS generation and participating in the TAM differentiation process (63).

DCs differentiated from monocytes have potent antigen presentation properties, promote T-cell activation, and play an important role in initiating and regulating immune responses (64). DC maturation is regulated by different types of stimuli. When immature DCs are stimulated by the pro-inflammatory cytokine IL-6 or the TLR ligand lipopolysaccharide, they are transformed into mature DCs that express CD80, CD86 and IL-6, and initiate effector T cell responses (65). In contrast, when DCs are stimulated by the regulatory factors IL-10, transforming growth factor beta (TGF-β), vitamin D3 and corticosteroids, they are transformed into tolerogenic DCs, which ultimately contribute to impaired differentiation of effector T cells and activation of Treg (66). The TME establishes an immunosuppressive state by inducing the differentiation of regulatory DCs and MDSCs, thus helping the tumor to escape immune surveillance (67). In addition, TGF-β and IL-10 secreted by tumor cells and TAM inhibit DC-mediated antigen presentation and adaptive immune responses (68). Ultimately, the concentration of ROS in the TME has an important role in regulating the cytotoxic or immunosuppressive effects of immune cells.

Mitochondria are the main ROS-producing organelles in cells, producing ROS and OXPHOS through ETC, which have a dual role in tumors: low levels are important cell signaling molecules, high levels cause oxidative damage and promote tumor development, and ROS balance is key to cell homeostasis.

In tumors, ROS drives cancer progression, and tumor cells can adapt to a high ROS environment, and also regulate the activation state of TME immune cells, such as damaging the stability of T cell-related complexes, synergizing with immunosuppressive cells to help build immune tolerance, and Lon protease is also involved in promoting mtROS production. At the same time, ROS affects a variety of immune cells: regulates the activity of NK cells and T lymphocytes, promotes MDSC differentiation, affects macrophage polarization and function, regulates DC maturation and antigen presentation, and ROS concentration in the TME plays an important role in regulating immune cell function.

### 2.3 Role of mtDNA in anti-tumor immunity

**Figure 4:**
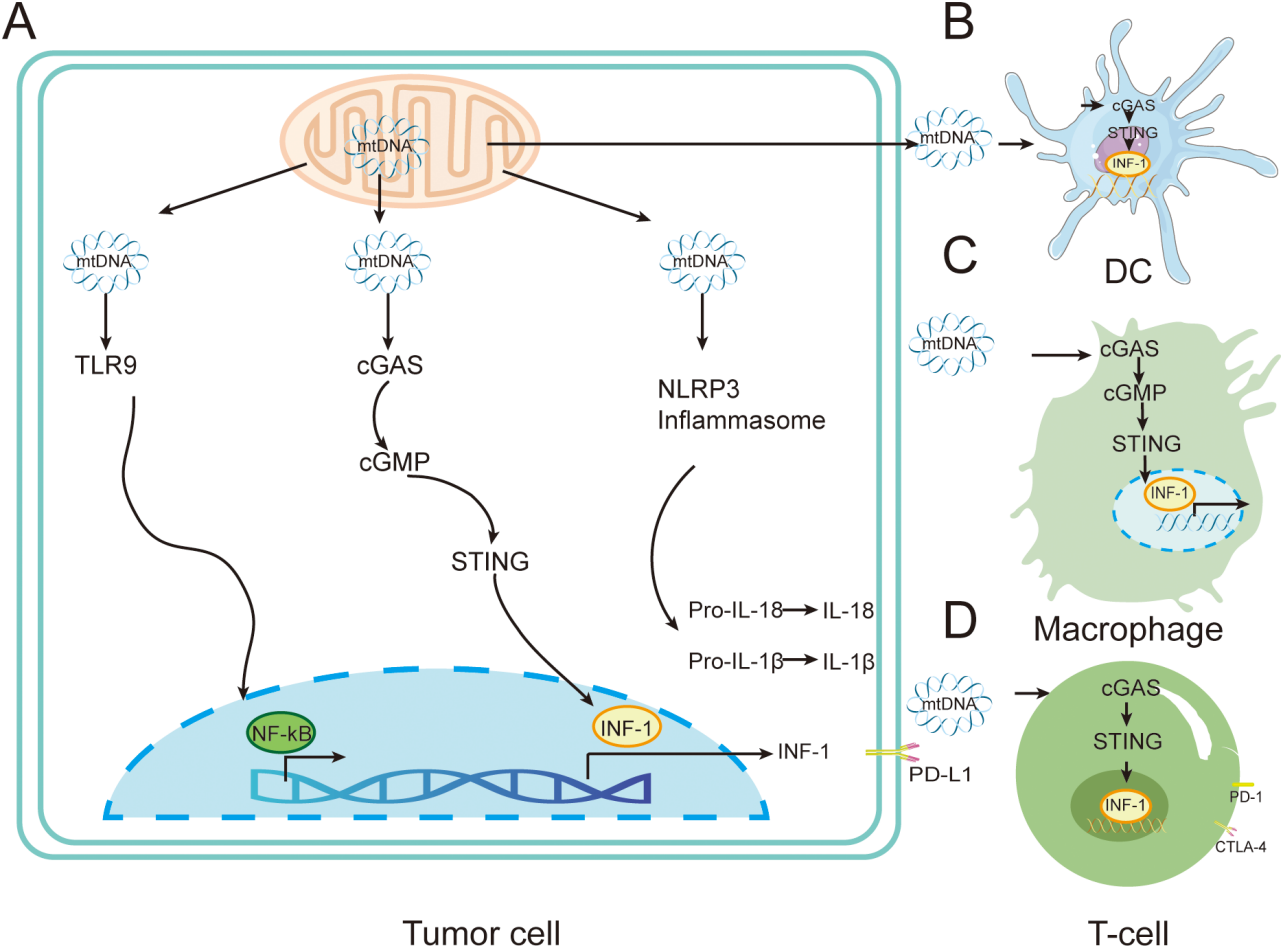
Immune regulation by mtDNA in tumors cells. **(A)** Mitochondria can release mtDNA in response to external or endogenous stress. The released mtDNA triggers various pro-inflammatory signaling pathways through TLR9, cGAS-STING, or through the cytoplasmic inflammasome NLRP3. **(B)** mtDNA released by tumor cells is transferred to DC cells, stimulating the activation of the cGAS-STING pathway and leading to the release of type I interferons. **(C)** mtDNA released by tumor cells is transferred to DC cells, stimulating the activation of the cGAS-STING pathway and leading to the release of type I interferons, inducing immunosuppressive M2 phenotype macrophages. **(D)** mtDNA released by tumor cells is transferred to T cells, stimulating the cGAS-STING pathway and leading to the release of type I interferons.

Genomic mutations in mitochondria are an important part of the cancer mutant genome, and mtDNA dysfunction and gene mutations are closely related to cancer development (69). Mitochondrial gene copy number abnormalities, aberrant gene expression and altered mtDNA epigenetic modifications frequently affect cancer development and malignant transformation by regulating cellular metabolism, ROS production and cell-cell interactions. Furthermore, the location and level of mtDNA gene mutations can confer different degrees of competitive advantages to cancer cells (70). mtDNA leaked into the cytoplasm by mitochondria during stress is an important source of DAMPs, and cytoplasmic mtDNA binds to and activates different DNA pattern recognition receptors, inducing strong intrinsic immune responses (71) (**Figure 4**).

When mitochondrial stress occurs, mtDNA can be activated by BAX/BAK-dependent mitochondrial outer membrane permeabilization (MOMP) or mitochondrial permeability transition pore (mPTP), and mtDNA can bind to and activate different DNA pattern recognition receptors to induce strong intrinsic immune responses (72). After release into the cytoplasm, mtDNA can be recognized by pattern recognition receptors such as cGAS, TLR9 and NLRP3, which activate downstream inflammatory signaling pathways.

When mtDNA leakage occurs, the cytoplasmic localized receptor cGAS recognizes mtDNA and induces generation of the second messenger 2´3´-cGAMP. Subsequently, cGAS activates endoplasmic reticulum-localized protein STING and mediates the downstream activation of the type I interferon (INF-I) signaling pathway and the associated inflammatory response (73). Studies have shown that cGAS-STING signaling activation has an important regulatory role in tumor immunity. Tumor-specific adaptive immune responses, including cytotoxic T-cell (CD8^+^ T-cell) activation, are dependent on INF-I signaling from APCs. Activation of INF-I is largely mediated by the cGAS-STING signaling pathway (74). mtDNA can enhance the function of Treg through the cGAS-STING signaling pathway, thereby suppressing tumor immunity and promoting the development of T lymphoma (75). Fatty acid binding protein 5 (FABP5) is one of the proteins that maintains mitochondrial stability in T cells. Thus, FABP5 inhibitors impair mitochondrial integrity and promote the release of mitochondrial DNA, thereby inducing interleukin 10 (IL-10) production via activation of the cGAS-STING signaling pathway. IL-10 facilitates T-lymphoma development by promoting Treg in the TME to suppress the viability of other T-cells (76). Mitochondrial DNA upregulates programmed cell death ligand 1 (PD-L1) and IDO-1 via the cGAS-STING signaling pathway, thereby inhibiting T cell function (77). CD47 blockade disrupts SIRPα-CD47 signaling, preventing lysosomal degradation of phagocytosed mtDNA in DCs, thereby enhancing cGAS sensing (78). Ionizing radiation can damage the mitochondria of tumor cells such as colon cancer, lung cancer and T lymphoma, resulting in the release of mtDNA, which is phagocytosed by DC cells and activates the cGAS-STING signaling pathway, enhancing the ability of DC cells to deliver antigens to CD8^+^ T cells, and ultimately enhancing tumor immunity (79). The ataxia telangiectasia mutated (ATM) protein detects DNA double-strand breaks and promotes DNA damage repair. Pharmacological inhibition of ATM (e.g., KU-55933) reduces mitochondrial transcription factor A (TFAM) expression in melanoma and breast cancer cells, promotes mtDNA leakage into the cytoplasm, activates the cGAS-STING signaling pathway and downstream cytokine production, and enhances lymphocyte infiltration into the TME, resulting in anti-tumor therapeutic effects (80). Activation of cGAS-STING signaling can also activate a variety of immune cells including DCs, macrophages, NK cells, CD4^+^ and CD8^+^ T cells by triggering the relevant natural immune signals, leading to reduction or even complete disappearance of a variety of tumors *in vivo (* 81).

TLR9 supports tumor cell growth and chemoresistance by recognizing the CpG structural domain of mtDNA which activates downstream MAPK and NF-κB signaling to promote the associated inflammatory responses (82). Notably, mtDNA leaking into the extracellular space can also be involved in the polarization and functional regulation of a variety of immune cells, including macrophages, DCs, and T lymphocytes, through the activation of TLR9 and cGAS-STING signaling in neighboring immune cells (83).

NLRP3, as a multicomponent protein complex in the cytoplasm, recognizes mtDNA leaking into the cytoplasm and activates downstream MAPK and NF-κB signaling (84). mtDNA activates NLRP3 inflammasome assembly via K^+^ efflux, leading to caspase-1-dependent IL-1β maturation (85).

Mitochondrial genome mutations constitute a significant portion of the mutation genome in cancer. Their functional impairments, mutations, copy number abnormalities, aberrant expressions, and alterations in epigenetic modifications can influence cancer development and malignant transformation by regulating cellular metabolism, ROS production, and intercellular interactions. Moreover, the specific locations and levels of these mutations can confer a competitive advantage to cancer cells. Under mitochondrial stress, mitochondrial DNA can be activated via specific mechanisms and released into the cytoplasm or extracellular space, where it can be recognized by receptors such as cGAS, TLR9, and NLRP3. Notably, cGAS activates STING upon recognition, mediating associated signaling pathways and inflammatory responses, which play a crucial role in tumor immunity—potentially inhibiting tumor immunity and promoting cancer, or enhancing tumor immunity and producing anti-tumor effects; it can also activate various immune cells to reduce tumors. TLR9 recognizes specific structural domains, supports tumor growth, enhances chemotherapy resistance, and contributes to the regulation of immune cell function. NLRP3, upon recognition, activates downstream signals, and mtDNA can induce the assembly of its inflammasome through potassium efflux.

### 2.4 Role of mitochondrial autophage in anti-tumor immunity

Tumor mitochondrial autophagy is a specialized autophagic process in tumor cells that selectively eliminates damaged or redundant mitochondria (86). This autophagy relies on pathways such as Parkin-PINK1, BNIP3/BNIP3L, and FUNDC1, serving as a key mechanism regulating mitochondrial homeostasis in tumor cells (87). Mitochondrial autophagy exhibits dual-sided effects: on one hand, it helps tumor cells adapt to hypoxic and nutrient-deprived microenvironments by eliminating damaged mitochondria, reducing ROS accumulation and mtDNA release, thereby maintaining metabolic balance for survival; on the other hand, its excessive activation reduces tumor cell immunogenicity, suppresses innate immune pathways like cGAS-STING, and facilitates immune evasion (88). Conversely, mitochondrial autophagy defects lead to mtDNA accumulation, activating immune responses resulting in the enhancement of antitumor immunity. Currently, targeted regulation of tumor mitochondrial autophagy has emerged as a promising therapeutic approach. By intervening in related pathways and synergizing with immune checkpoint blockade, it can improve tumor treatment efficacy.

Autophagy is a key mechanism supporting the maintenance of activated states and antitumor functions in CD8^+^ T cells. Following tumor antigen recognition by the T cell receptor (TCR), basal autophagy activity is triggered: it degrades intracellular surplus proteins to release amino acids and other metabolic substrates, providing energy for CD8^+^ T cell proliferation while maintaining organelle homeostasis. Simultaneously, it regulates immunological synapse formation, promotes TCR signaling activation, and drives CD8^+^ T cells from a quiescent to an effector state.

However, the hypoxic microenvironment of the TME can induce autophagy via HIF1α, downregulating MHC-I molecule expression and thereby reducing CD8^+^ T cell cytotoxicity (89). Similarly, CXCL1 mediates MHC-I degradation via autophagy in colorectal cancer (CRC), while the oncogene PACSIN1 promotes MHC-I lysosomal degradation through autophagy in gastric cancer, both inhibiting antigen presentation and CD8^+^ T cell infiltration to drive immune escape (90, 91). Furthermore, defects in autophagy-related genes (ATG) significantly impact CD8^+^ T cell function: Atg5/Atg7 deficiency enhances CD8^+^ T cell infiltration and IFN-γ secretion, while Atg4/Atg5 knockdown upregulates MHC-I expression and antigen presentation in lung cancer cells; while Atg7 deficiency suppresses tumor cells through metabolic promotes CD8^+^ T cell accumulation in the colonic lamina propria (92–94). Clinical studies reveal that LC3B expression in hypopharyngeal squamous cell carcinoma (HSCC) positively correlates with CD8^+^/CD39^+^ T cell infiltration, while LC3B deficiency in breast cancer reduces CD8^+^ T cell infiltration and increases FOXP3^+^ Treg/CD68^+^ macrophage numbers, suggesting autophagy influences tumor prognosis by regulating CD8^+^ T cell infiltration (95).

Notably, autophagy modulates CD8^+^ T cell function by regulating immune checkpoints and cytokines: in CRC, CTSS upregulates PD-L1 via autophagy and reduces CD8^+^ T cell infiltration (96). In acute myeloid leukemia (AML), C/EBPα DM alleviates CD8^+^ T cell immunosuppression by inhibiting autophagy-associated IL-1β secretion (97). Combining autophagy inducers with chemotherapeutic agents specifically activates CD8^+^ T cell-dependent anticancer immunity, suggesting that targeting autophagy may serve as a potential strategy to enhance CD8^+^ T cell antitumor function.

In the TME autophagy provides essential survival support for Tregs by degrading intracellular glycogen, damaged proteins, and releasing metabolic substrates from mitochondria. Simultaneously, mitochondrial autophagy clears hypoxia-induced damaged mitochondria, reduces ROS accumulation, and prevents premature Treg apoptosis. Autophagy stabilizes Foxp3 transcription factor expression in Tregs, promotes synthesis of inhibitory cytokines like IL-10 and TGF-β, and enhances their immunosuppressive effects on CD8^+^ T cells. CAFs in the TME can activate Treg expansion through antigen-dependent and autophagy-dependent pathways by forming immune synapses with Tregs (98). UNC-51-like kinase 1 (ULK1), as an autophagy-activating molecule, is a key candidate target for regulating Treg function (99). Clinical studies reveal abnormally elevated CD39^+^ Treg levels in patients with autophagy genetic defects, and these CD39^+^ Tregs show low expression of autophagy-related genes NEFL and PLAC8 (100). Furthermore, defects in the GTPase-activating regulator RGS1 disrupt Treg metabolism and autophagy via the FOXP3-c-MYC axis, diminishing their immunosuppressive capacity (101). This suggests autophagy is a core pathway for maintaining Treg function.

In the TME, autophagy regulates TAM polarization. In undifferentiated pleomorphic sarcoma (UPS), COLVI induces CD8^+^ T cell dysfunction by inhibiting T cell autophagy while promoting TAM M2 polarization and VEGF/TGF-β secretion, thereby facilitating tumor angiogenesis (102). Oxidative stress induces tumor cells to release KRAS(G12D), which is packaged into exosomes via autophagy-dependent ferroptosis and induces M2 polarization in macrophages through the AGER-STAT3 axis (103).

Conversely, under stimuli like chemotherapy drugs, autophagy induces M1 polarization in TAMs. Autophagy inhibition enhances pro-inflammatory effects associated with M1 polarization by regulating macrophage migration inhibitory factor (MIF) secretion via ROS; while mTOR signaling inhibition reduces M2-type TAMs and MDSCs in the TME by downregulating autophagy, simultaneously upregulating CD8^+^/CD4^+^ T cells (104). This suggests autophagy serves as a critical regulatory node in TAM polarization.

Hypoxia and immunosuppressive factors in the TME can impair DC function, whereas autophagy maintains DC activity through multiple mechanisms: on one hand, it degrades senescent mitochondria and damaged proteins within DCs, preserving energy homeostasis and reducing ROS-induced apoptosis to safeguard DC numbers; on the other hand, it degrades tumor antigens through autophagosome-lysosome fusion, generating antigenic peptides for presentation via MHC class I/II molecules. Crucially, it facilitates antigen shunting to the MHC class I pathway during cross-presentation, efficiently activating CD8^+^ T cells (105).

Autophagy also regulates DC maturation and cytokine secretion: activating autophagy promotes DC expression of co-stimulatory molecules like CD80 and CD86 while secreting IL-12, enhancing immune activation. Conversely, autophagy defects leave DCs in an immature state, even secreting TGF-β to exacerbate immune suppression (105). Furthermore, ROS-dependent endoplasmic reticulum stress in tumor cells suppresses DC surface calretinins exposure via autophagy, diminishing their maturation capacity and IL-6 secretion, thereby inhibiting CD4^+^/CD8^+^ T cell proliferation (106). High Mobility Group Box 1 (HMGB1) inhibits DC apoptosis via the JNK-autophagy axis, contributing to colon cancer cell immune evasion. This suggests autophagy is a core regulatory mechanism for DC-mediated antitumor immunity (106).

Autophagy enhances NK cell antitumor activity by regulating the synthesis and recognition of killing molecules. Disrupting the interaction between ATG7 and phosphorylated FOXO1 in the cytoplasmic solute of immature NK cells blocks autophagy, a process critical for NK cell maturation. Activating autophagy may support the maturation of NK cells and other ILCs exhibiting anticancer activity (107). In the B16-F10 melanoma model, the autophagy-critical gene Beclin1 induces substantial infiltration of functional NK cells into the tumor bed by activating the MAPK8/JNK-JUN/c-Jun signaling pathway, significantly inhibiting tumor growth (108). This further confirms autophagy's positive regulatory role in NK cell antitumor function. Targeting Becn1 to inhibit autophagy significantly restores the levels of serine protease GZMB/granzyme B within target cells under hypoxic conditions and induces tumor regression *in vivo* by promoting NK cell-mediated tumor cell killing (109, 110).

Autophagy defects impair MDSC lysosomal degradation, upregulate MHC-II molecule expression, thereby activating tumor-specific CD4^+^T cells and reducing tumor volume; conversely, in multiple myeloma (MM), MDSCs induce tumor cell autophagy via AMPK phosphorylation, upregulating MCL-1/BCL-2 expression to enhance MM cell survival (111). Mechanistically, glycolysis inhibits CCAAT enhancer-binding protein β (CEBPβ) subtype LAP expression via the AMPK-ULK1-autophagy axis, thereby regulating G-CSF/GM-CSF secretion to support MDSC development (111). While the lysosomal inhibitor LCL521 disrupts autophagy by activating cathepsin B/D, inducing endoplasmic reticulum stress in MDSCs and promoting their death, providing a basis for therapies targeting MDSC autophagy (112).

## 3 Mitochondrial targeting strategies

**Figure 5:**
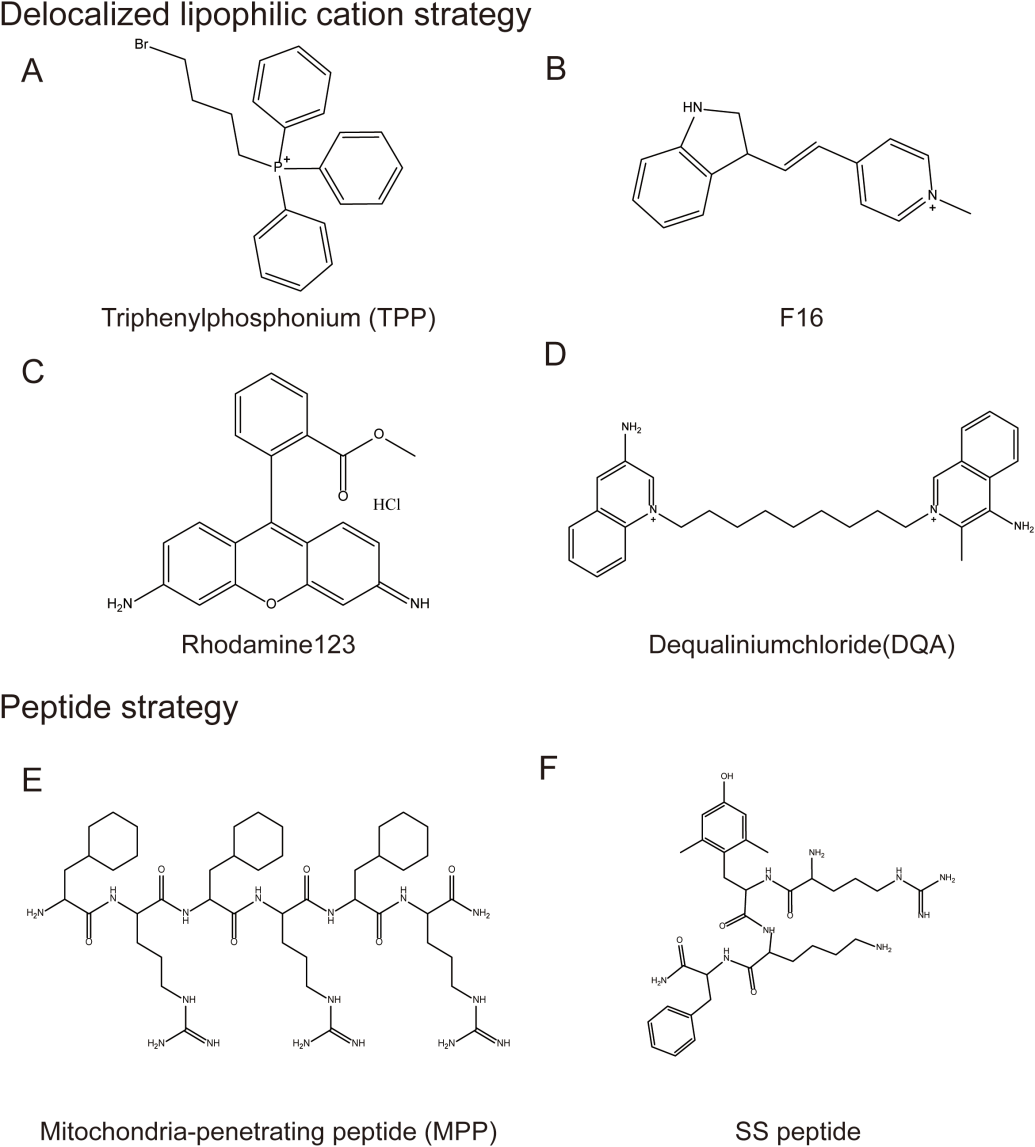
Mitochondrial-targeted structural modification strategies. Typical Mitochondrial-targeted structural modification strategies: 1. Delocalized lipophilic cation strategy, including **(A)** Triphenylphosphonium, **(B)** F16, **(C)** Rhodamine123, **(D)** Dequaliniumchloride; 2. Peptide strategy including **(E)** Mitochondria-penetrating peptide and **(F)** SS peptide.

Mitochondria have great potential as therapeutic targets. Drug entry into cells requires passage through several lipid bilayers, especially the inner mitochondrial membrane which is highly selective for molecular traversal, which is the reason why mitochondria-targeted drugs are difficult to deliver (113). Currently, several methods have been developed to enable targeted drugs to break through the lipid bilayer into the mitochondria, such as delocalized lipophilic cation (DLCs) modification and mitochondria-targeted drug delivery technologies (114) (**Figure 5**).

### 3.1 Mitochondrial targeting based on DLC modification

DLCs are a class of compounds that can penetrate the lipid bilayer and accumulate in mitochondria, due to the high mitochondrial membrane potential of tumor cells. These compounds can be used for targeted delivery of drugs to mitochondria of tumor cells by covalently linking them with small molecules. DLCs identified in current studies include triphenylphosphine (TPP^+^) and its derivatives, F16, rhodamine analogs, and dequaliniumchloride (DQA).

#### 3.1.1 TPP and its derivatives

**Figure 6:**
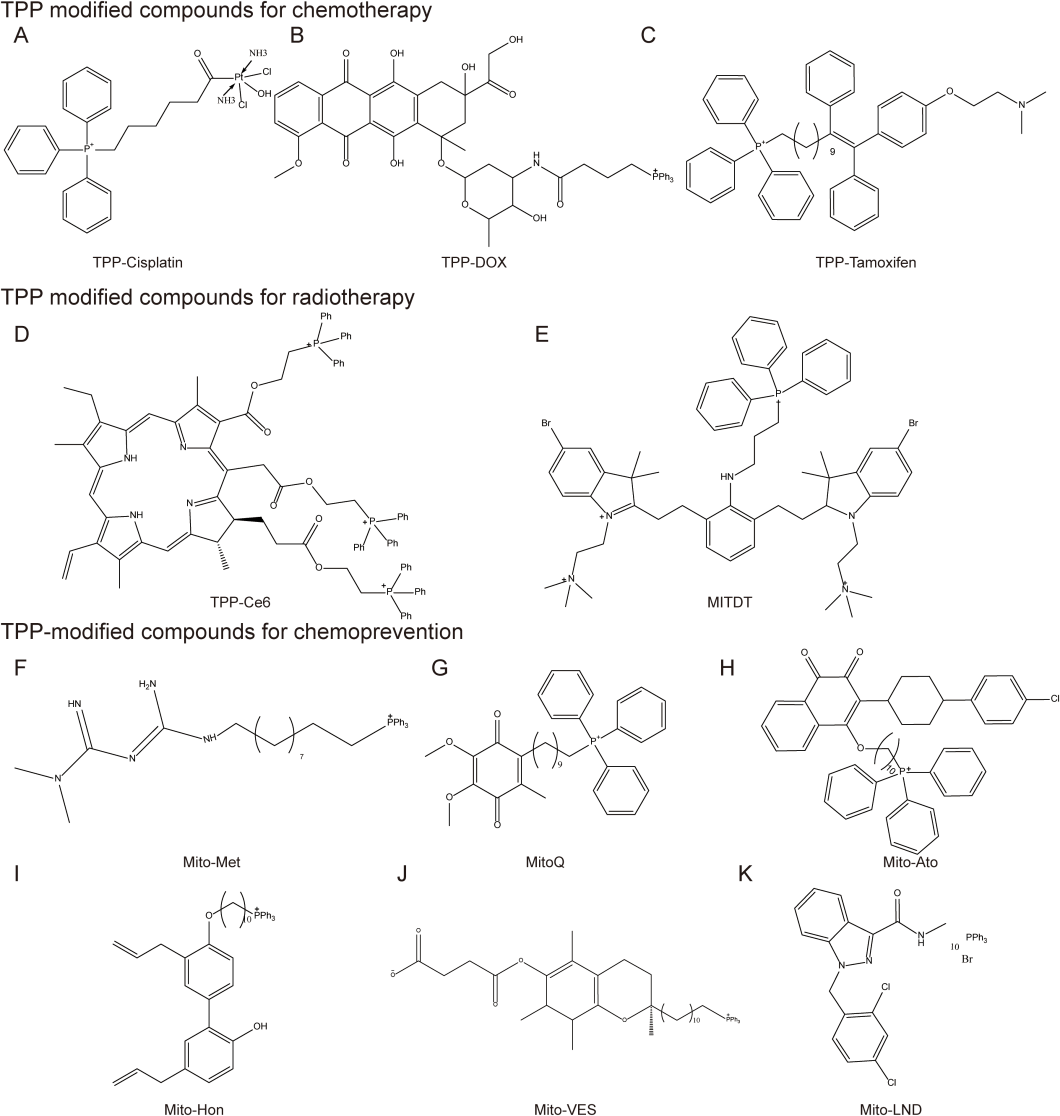
Mitochondrial-targeted triphenylphosphine compounds. TPP modified compounds for chemotherapy include **(A)** TPP-Cisplatin, **(B)** TPP-DOX, **(C)** TPP-Tamoxifen; TPP modified compounds for radiotherapy include **(D)** TPP-Ce6 **(E)** MITDT; TPP-modified compounds for chemoprevention, including **(F)** Mito-Met, **(G)** MitoQ, **(H)** Mito-Ato, **(I)** Mito-Hon, **(J)** Mito-VES, and **(K)** Mito-LND.

The chemical structure of TPP contains three phenyl groups, which makes it highly lipid-soluble (115). At the same time, the positively charged phosphorus ions can be delocalized to the three benzene rings, allowing them to pass smoothly through the lipid bilayer (116). TPP drives drug accumulation within mitochondria due to the the negative mitochondrial membrane potential (ΔΨm) (117) (**Figure 6**).

**Figure 7:**
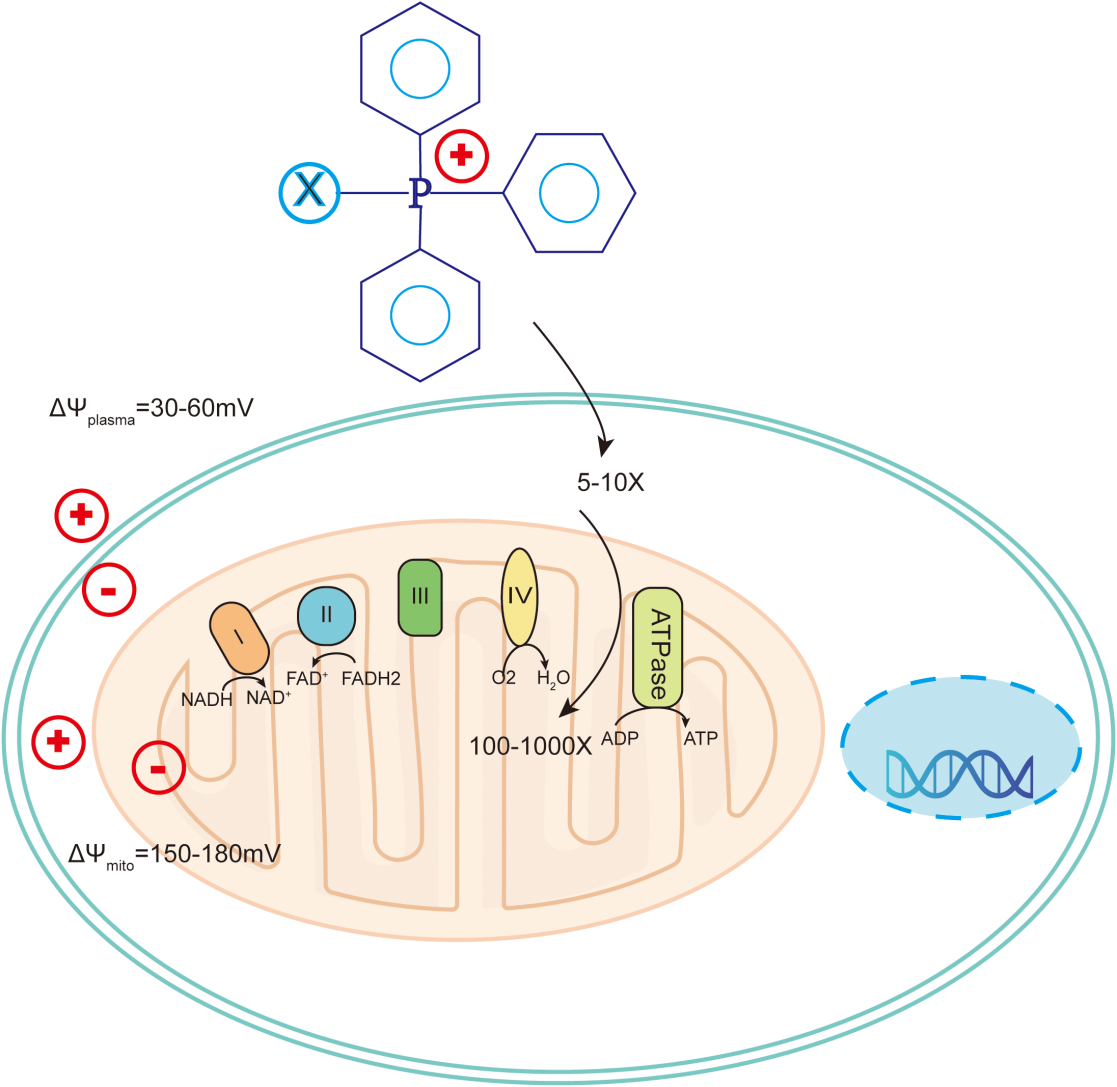
Mechanism of triphenylphosphine targeting to mitochondria in tumor cells. Cancer cells have more hyperpolarized membranes than normal cells, helping to drive uptake of TPP+-conjugated compounds by up to 100-1000-fold. .

Chemotherapy drugs have long been the mainstay of therapy for many cancer types. However, severe side effects, low bioavailability, poor stability, and acquired drug resistance limit their clinical application. Mitochondria-targeted monofunctional platinum complexes can accumulate in the mitochondria, induce significant changes in mitochondrial ultrastructure and membrane, release cytochrome c(Cytc) from mitochondria, and disrupt mitochondrial OXPHOS and glycolysis (118). Paclitaxel (PTX) modified with TPP cations reduced the decrease in ΔΨm and significantly inhibited the growth of MCF-7 cells. Doxorubicin (DOX) resistance is a common problem in cancer treatment (119). Addition of TPP to DOX-PLGA/CPT nanoparticles leads to effective mitochondrial localization of DOX-PLGA/CPT, releases DOX to target mtDNA, induces tumor cell apoptosis and overcomes DOX resistance in MCF-7/ADR breast cancer cells (120) (**Figure 7**).

Photodynamic therapy (PDT) is being used to treat some cancers, and it has become a promising approach for the treatment of malignant brain tumors. Mitochondria-targeted triphenylphosphine can enhance PDT efficacy in brain cancer. The TPP-conjugated photosensitizer chloramphenicol e6 (Ce6) selectively accumulates in mitochondria, colocalizes with 88% of mitochondria, and has potent cytotoxic activity, thereby significantly enhancing PDT efficacy (121, 122). The PDT effect of the mitochondria-targeting photodynamic therapeutic (MitDt) agent is amplified after laser irradiation because mitochondria are susceptible to ROS, triggering apoptotic anticancer effects (123). TPP-modified photosensitizer zinc phthalocyanine (ZnPc) selectively accumulates in mitochondria, showing excellent mitochondrial targeting for ROS-activated chemotherapy and PDT (124). TPP-modified liposomes encapsulating black phosphorus (BP) and calcium peroxide (CaO_2_) accumulate in tumor mitochondria and are activated by near-infrared (NIR) laser irradiation to generate abundant PO4^3-^ and Ca^2+^ to accelerate *in situ* mitochondrial mineralization, leading to mitochondrial dysfunction and cancer cell death (125).

Radiotherapy has been an important form of cancer treatment for many years. TPP can significantly increase the efficacy of radiation enhancers and improve the effect of radiotherapy. Smaller doses of radiation (4Gy vs standard 12Gy) can be given in combination with TPP-based PDT to control tumor growth, reducing radiation side effects (126). 4-hydroxy-2,2,6,6-tetramethyl-1-oxy-piperidin(Tempol) coupled with TTP enhances X-irradiation-induced germ cell death, reduces basal ΔΨm and inhibits X-ray-induced increase in ATP production (127).

Photothermal therapy (PTT) is a new non-invasive tumor treatment that uses photothermal agents (PTA) to convert light energy into heat energy to kill tumor cells under irradiation with external light sources such as NIR light. Micro-nanoparticles loaded with TPP and S-nitrosothiol can release NO generated by surface overheating and elicit PTT upon NIR laser irradiation. The released NO can also destroy collagen fibers by activating matrix metalloproteinases (MMPs), thereby loosening the dense extracellular matrix (ECM) to enhance immune cell infiltration. The highly toxic reactive nitrogen species (RNS) peroxynitrite (ONOO^-^) is produced, resulting in mitochondrial damage and induction of cell apoptosis (128). Nanoparticles with core-shell-disulfide-shell nanoparticles burst in the high GSH environment of tumors to achieve targeted drug release. The loaded DOX can quickly enter mitochondria, subsequently destroying mitochondrial DNA and leading to cell apoptosis. The synergistic effect of PTT and chemotherapy targeting mitochondria significantly enhances cancer treatment (129). The heat stress-damaged mitochondria produced can cause ICD in tumor cells, release damage-related factors, reactivate the immune response of macrophages against tumor cells, and effectively activate tumor-associated macrophages to fight against tumor cells (130).

TPP conjugates enhance the accumulation of chemopreventive agents in tumor cell mitochondria, enhancing efficacy and reducing toxicity to normal tissues (131). Metformin (Met), a commonly used hypoglycemic drug, has certain mitochondria-targeting effects and anti-tumor ability, but its clinical performance is not ideal. In pancreatic ductal adenocarcinoma (PDAC) cell lines, the IC_50_ concentration required to inhibit proliferation by Mito-Met is nearly 1,000 times lower than that of Met (132). Mito-Met induces superoxide (O2•^-^) production through complex I, inducing ROS-disrupting membrane potential, and activating calcineurin- and Cn-dependent retrograde signaling pathways in multiple cells (133). Atovaquone(ATO), an antimalarial drug, was discovered to have anti-tumor potential in the form of TPP-modified and PEGylated mitochondrial-targeted ATO (Mito-(PEG)n-ATO). Mito-ATO analogs inhibit mitochondrial complex I and complex III-induced OXPHOS in human pancreatic and brain cancer cells. Combined use with inhibitors of monocarboxylate transporters (MCT), Krebs cycle redox metabolism, or glutaminolysis, the Mito-ATO analogs can synergistically eliminate tumor cell proliferation (134). TPP mitochondrial targeting increases the drug's involvement in metabolic processes within mitochondria, inhibits tumor cell development, and promotes tumor cell death (135).

TPP has a high fat-soluble transportable positively charged phosphorus ion, which can drive the accumulation of drugs in mitochondria with the help of mitochondrial negative membrane potential. Drugs such as metformin and atropine can be used in chemotherapy to increase efficacy and overcome resistance, in radiotherapy to increase efficacy and reduce side effects, and in PTT to aid the initiation of treatment and destruction of ectoplasm, induction of apoptosis. TPP-derived compounds exhibit good antitumor activity, but there are few clinical studies to verify their anticancer efficacy, and further clinical studies are needed in the future (117). Although the TPP cation itself has low toxicity, some of the TPP-derived compounds administered systemically have non-specific toxicity, and the current strategy is mainly to modify the structure of TPP cations, encapsulate the modified compounds in liposomes, and reduce the toxicity of TPP cations, enhance its targeting selectivity to tumor cells and mitochondria to reduce toxicity (131).

#### 3.1.2 F16

F16 is a delocalized DLC that can target and aggregate in the mitochondrial matrix of tumor cells. F16 induces mPTP opening by inhibiting the interaction between mitochondrial inner membrane adenine nucleotide translocase (ANT) and cyclophilin D. F16 can form conjugates with other substances to enhance anti-tumor effects. At the same time, the reduced availability of intracellular adenosine 5'-triphosphate induced by the uncoupling effect of F16 is the main factor in the enhanced cytotoxicity mediated by F16 (136). F16 conjugates show higher cytotoxicity at low doses, and F16 conjugates initiate cell cycle arrest at the G0/G1 phase leading to mitochondrial dysfunction and excessive production of ROS, thereby inducing apoptosis (137–139). The F16-modified compounds accumulate in cancer cell mitochondria to depolarize ΔΨm, increase ROS and attack mtDNA, effectively killing cancer cells and overcoming multi-drug resistance (140, 141). Fluorescent mitochondria-targeted organic arsenic accumulates in mitochondria and inhibits the activity of pyruvate dehydrogenase complex (PDHC), leading to ATP synthesis inhibition and heat production disorders. The inhibition of respiratory chain complexes accelerates mitochondrial dysfunction and causes cell apoptosis (142).

F16 can also be used for fluorescence imaging of mitochondria. CyM is a multifunctional organic biological probe that can facilitate NIR imaging and PDT *in vivo* and *in vitro (* 143). There are two F16 isomers that can specifically display mitochondria in the green and red channels, respectively, due to their unique fluorescence properties, providing new ways of studying mitochondrial targeting by F16. The above studies suggest that F16 and its derivatives can be of great value in cancer treatment and tumor imaging. However, the clinical application of F16 is limited by its toxicity to normal cells (144). Therefore, scientists have focused on enhancing the selectivity of F16 and its derivatives for tumor cells, and future research will likely uncover new drugs that can specifically target tumor cell mitochondria and reduce toxic effects on normal cells.

In summary, F16 acts as a DLC that can be targeted to cluster in the mitochondrial matrix of tumor cells, and its conjugate can depolarize δψm, increase Ros to attack mtDNA to kill cancer cells, and overcome multiple drug resistance. F16 can also be used for mitochondrial fluorescence imaging. 

#### 3.1.3 Rhodamine

Rhodamine is an organic fluorescent dye based on xanthene that can be substituted with different 3- and 6-amino groups. It has a darker color and stronger fluorescence signal. Rhodamine can penetrate the cell membrane and selectively stain the mitochondria of living cells. Rhodamine dyes have photophysical properties such as high fluorescence quantum yield, high molar extinction coefficient and good water solubility, and low biological toxicity, making them attractive for wide use as biomarkers and fluorescent probes.

Rhodamine conjugates can be delivered to tumor mitochondria and functional proteins through organic cation transporters, improving their tumor inhibitory effects. Rhodamine B mitochondria-targeted multi-drug nanoparticles focus on mitochondrial stress-induced ICD to improve their therapeutic effect on treatment of ovarian cancer (145). Centella asiatica-rhodamine B conjugates are highly cytotoxic to human tumor cell lines, affect cell apoptosis, and can overcome resistance to chemotherapeutic drugs (146). Rhodamine B-conjugated oleanolic acid derivatives (RhodOA) reduce tumor cell viability, reduce cell migration and disrupt mitochondrial function (147). Hybrid peptide-fused rhodamine B increases anticancer activity by up to 37.5 fold, targeting the nucleus and triggering apoptosis to enhance anticancer cell activity (148). Enrichment of rhodamine B-modified catalase in cancer tissues can effectively inhibit mouse xenograft human lung tumors (149). Differences in ΔΨm and ATPase sensitivity in tumor cells contribute to the selective cytotoxicity of rhodamine123 against certain cell types *in vitro (* 150). Mitochondrial targeting by rhodamine enhances the preferential cellular uptake of paclitaxel and SN-38 in cancer cells by 2–3 fold (151). Multi-walled carbon nanotubes (MWCNTs) with mitochondria-targeted fluorescent rhodamine-110 colocalize 80% with mitochondria and exhibit superior efficacy to drugs without PtBz (152). Ciacic acid-rhodamine 101 conjugates induce proliferation or growth arrest of MDA-MB-231 breast cancer cells at low doses and induce apoptosis at higher doses (153).

Rhodamine can significantly improve the effect of PDT on tumors and is by itself a potential PDT agent. The combination of rhodamine organic dyes and luminescent transition metal centers exhibits low cytotoxicity, increases tumor cell uptake, and enhances antitumor efficacy (154). Rhodamine 6G-based organic salts are stable under physiological conditions and show excellent fluorescence photostability. More importantly, they have tunable chemotherapeutic properties. Rhodamine fluorescent groups synthesized from Rh-6G and amines show pH-dependent anticancer bioactivity and trigger cell apoptosis through mitochondrial pathways, showing anticancer bioactivity in bladder cancer (155). Rhodamine 6G-based organic salts can produce nanoparticles that are toxic to cancer cells but not normal cells (156). The apoptotic index of Dasatinib (DST) contained nanoparticles is 7.5 times higher than that of free DST and is non-toxic to normal cells (157). Rhodamine-mediated novel supramolecular assemblies can efficiently capture phosphorescence energy transfer (PET) processes and have potential applications in delayed fluorescence cell imaging (158). Mitochondria-targeted silicon rhodamine-based photosensitizer (SiR-PXZ) can be rapidly taken up by mitochondria and effectively induce cancer cell apoptosis, showing excellent anti-tumor effects and potential value in photodynamic cancer therapy (159). Burst-specific PDT in mitochondria by the rhodamine derivative UCNP-GQD/TRITC induces a sharp drop in ΔΨm, thereby irreversibly initiating tumor cell apoptosis (160).

Rhodamine, with its ability to penetrate cell membranes and selectively stain mitochondria in living cells, is often used as a biomarker and fluorescent probe, its conjugates can target tumor mitochondria to inhibit tumor cells, overcome drug resistance and enhance anticancer drug uptake through a variety of mechanisms. Rhodamine can improve the effect of tumor PDT and is a potential PDT agent, showing potential value in fields such as photodynamic cancer therapy and delayed fluorescence cell imaging.

#### 3.1.4 DQA

DQA is a DLC with two positive charge centers. It can selectively accumulate in mitochondria driven by transmembrane potential, allowing anti-tumor drugs to target mitochondria in tumor cells. DQA-coupled FMPSi-Cis@GO targets mitochondria in cancer cells and destroys their function (161). DQA-containing micelles deliver DOX to the mitochondria and nucleus of tumor cells, significantly inhibiting the growth of DOX-resistant tumors without obvious systemic toxicity (162). Amphiphilic polymer GC-DQA nanoparticles were synthesized as carriers to efficiently deliver curcumin to mitochondria (163). The emulsion of DQA and α-tocopheryl succinate (α-TOS) targeting mitochondria has good stability and can effectively target mitochondria and inhibit the growth of HeLa cells (164).

DQA modification destroys mitochondrial structure and induces cell death by generating ROS and dissipating ΔΨm. DQA causes loss of mitochondrial transmembrane potential, O2*^-^ accumulation and ATP depletion in this tumor cell line, alters mitochondrial function and induces cell death (165). Hinokiflavone (HF) hybrid micelles increase ROS levels, reduce ΔΨm, and induce mitochondria-mediated apoptosis (166). DQA chloride vesicles (HPS-DQAsomes) of DOX increase cytotoxicity to MCF-7/ADR cell lines, can target the delivery of therapeutic agents to mitochondria and induce mitochondria-driven apoptosis (167). DQA- polyethylene glycol (PEG)-modified resveratrol liposomes DLS (Res) selectively accumulate in mitochondria, inducing cytotoxicity of cancer cells by generating ROS and dissipating ΔΨm (168).

DQA is an inhibitor of apoptotic proteins that can directly inhibit the activity of caspases, regulate apoptosis through multiple pathways, and promote the degradation of Bcl-2 as an E3 ligase, thereby exerting anti-tumor effects. DQA hybrid micelles enhance the uptake of paclitaxel by drug-resistant breast cancer cells. Induction of tumor cell apoptosis is related to the activation of pro-apoptotic proteins Bax, Cytc, caspases-3, 9 and the inhibition of Bcl-2 and Mcl-1 (169). Targeted lonidamine liposomes selectively accumulate in mitochondria of drug-resistant A549 lung cancer cells, dissipate ΔΨm, open mitochondrial permeability transition pores, and release Cytc through translocation. A cascade of caspases 9 and 3 reactivity is initiated, which activates the pro-apoptotic Bax protein and inhibits the anti-apoptotic Mcl-1 protein, thereby enhancing cytotoxicity by acting on mitochondrial signaling pathways (170). The development of targeted resveratrol liposomes modified with DQA-PEG (2000)-DSPE on the liposome surface significantly enhance cellular uptake and selectively accumulate in mitochondria. They induce apoptosis in non-resistant and resistant cancer cells by dissipating ΔΨm, releasing Cytc, and increasing the activity of caspases 9 and 3 (171).

DQA can be combined with other drugs to exert a wide range of anti-tumor activities through targeting of mitochondria and is a safe and economical cancer treatment. However, the existing combination of nanomaterials and drugs has not yet achieved breakthrough results, and further research is needed on the role of DQA in targeting mitochondria and its synergistic effect with other drugs.

Due to the high mitochondrial membrane potential of tumor cells, DLC can penetrate the lipid bilayer to accumulate mitochondria, and is often covalently linked to drugs for targeted delivery, mainly including TPP and its derivatives, F16, rhodamine, and DQA. TPP and its derivatives are highly lipid-soluble and positively charged, which can help the accumulation of mitochondrial cells of drugs, enhance the efficacy of chemotherapy, PDT, radiotherapy, PTT, etc., and can also improve the effect of chemopreventive agents. F16 targets the mitochondrial stroma and induces mPTP opening, and its conjugates enhance cytotoxicity and aid tumor imaging, but are toxic to normal cells. Rhodamine can stain mitochondria, and the conjugate can enhance tumor suppression and improve PDT effect. DQA has two positive charge centers that help drugs target mitochondria, disrupt mitochondrial function, induce cell death, and can be combined with other drugs to fight tumors.

### 3.2 Peptide targeting sequences

#### 3.2.1 Mitochondria-penetrating peptide

MPPs are synthetic mitochondrial localization peptides composed of 4 to 16 amino acids, containing cationic and hydrophobic residues. Similar to DLC, MPPs can finely regulate the localization of mitochondria by changing lipophilicity and charge, and have a significant inhibitory effect on growth of tumor cells *in vivo* and *in vitro (* 172).

MPP-modified DOX copolymers can promote cell apoptosis and inhibit tumor metastasis by destroying mitochondria, inhibit the growth of breast cancer 4T1 cells *in vivo*, and overcome tumor resistance (173). MPP-modified DOX significantly enhances drug accumulation in mitochondria by 11.6 fold, resulting in a significant increase in ROS generation and a decrease in the production of ATP that can inhibit drug efflux and the growth of drug-resistant cancer cells (174). Nanoparticles (NPs) consisting of membrane-permeable peptide amphiphiles (MMPA) and small interfering RNA (siRNA) can specifically accumulate in mitochondria and inhibit tumor growth by inhibiting ATP production and repolarizing TAMs (175). Nanocomplexes of mitochondrial-penetrating peptides mtCPP1 and PepFect14 affect biological functions in cytoplasm and mitochondria and can effectively target mitochondrial genes (176). Poly(lactide-co-glycolide) (PLGA) conjugates with 6-mer mitochondrial penetrating peptides (MPP) can be used for mitochondrial targets without cytotoxicity. DOX modified with mitochondrial penetrating peptides (MPP) delivers the drug to cancer cell mitochondria, mediating apoptosis and enhancing therapeutic outcomes for multidrug resistant tumors (177). DOX modified with MPP promotes apoptosis and inhibits tumor metastasis by disrupting mitochondria (178).

Compared with DLC, MPP may have more potential as a ligand targeting mitochondria due to its advantages including good biocompatibility and low toxicity. Molecules modified by MPP include RNA, DNA and proteins which can be exploited for human cancer treatment (179).

MPP can regulate lipophilicity and charge to achieve mitochondrial localization, promote apoptosis, inhibit tumor growth and metastasis, and overcome drug resistance. Compared with DLC, MPP has the advantages of good Biocompatibility and low toxicity, and has the potential to be a mitochondrial targeting ligand. Its modified RNA, DNA and protein molecules can be used for human cancer treatment.

#### 3.2.2 Szeto-Schiller peptides

Szeto-Schiller (SS) peptides are usually composed of four positively charged amino acids and are a new small peptide targeting strategy. They can specifically target mitochondrial cardiolipin, enhance mitochondrial plasticity and re-establish optimal bioenergetics.

SS peptides can reduce mtROS production, inhibit the opening of mitochondrial permeability transition pores, and have significant effects in preventing oxidative stress or inhibiting mitochondrial ETC-induced cell apoptosis and necrosis (180). The peptide SS-31 specifically localizes in the mitochondrial inner membrane by interacting with cardiolipin and can be used in the treatment of patients with abnormal ΔΨm (181). The SS-31-modified DOX-loaded liposome delivery system LS-DOX can effectively cross the blood brain barrier (BBB) to target gliomas, and mitochondrial targeting of SS-31 can enhance cellular uptake (182). SS-31 selectively binds to cardiolipin through electrostatic and hydrophobic interactions. By interacting with cardiolipin, SS-31 prevents cardiolipin from converting Cytc to peroxidase while protecting its electron-carrying function. Therefore, SS-31 protects the structure of mitochondrial cristae and promotes OXPHOS (183). Similarly, with excellent mitochondrial targeting ability, SS-20 peptide modification is a promising strategy for mitochondria-targeted drug delivery systems (184). Conjugation of α-TOS with SS-20 achieves delivery to mitochondria, increases ROS generation, opens the mitochondrial permeability transition pore, reduces ΔΨm, and promotes cell apoptosis (185). SS peptides are therefore expected to be studied more extensively in the future as strategic molecules for targeting cancer.

Peptide targeting sequences mainly include mitochondrial penetrating peptides (MPPs) and Stuart Schiller (SS) peptides. MPP is composed of 4–16 amino acids, contains cations and hydrophobic residues, can regulate lipophilicity and charge to locate mitochondria, can enhance the accumulation of mitochondria, promote apoptosis of tumor cells, inhibit metastasis and drug resistance, and has good biocompatibility and low toxicity, and the modified molecule can be used in cancer treatment. SS peptides often contain four positively charged amino acids, which can target mitochondrial cardiolipids, reduce mtROS production, inhibit the opening of mitochondrial permeability transition pores, and protect mitochondrial structure and function.

### 3.3 Mitochondria-targeted drug delivery technology

**Figure 8:**
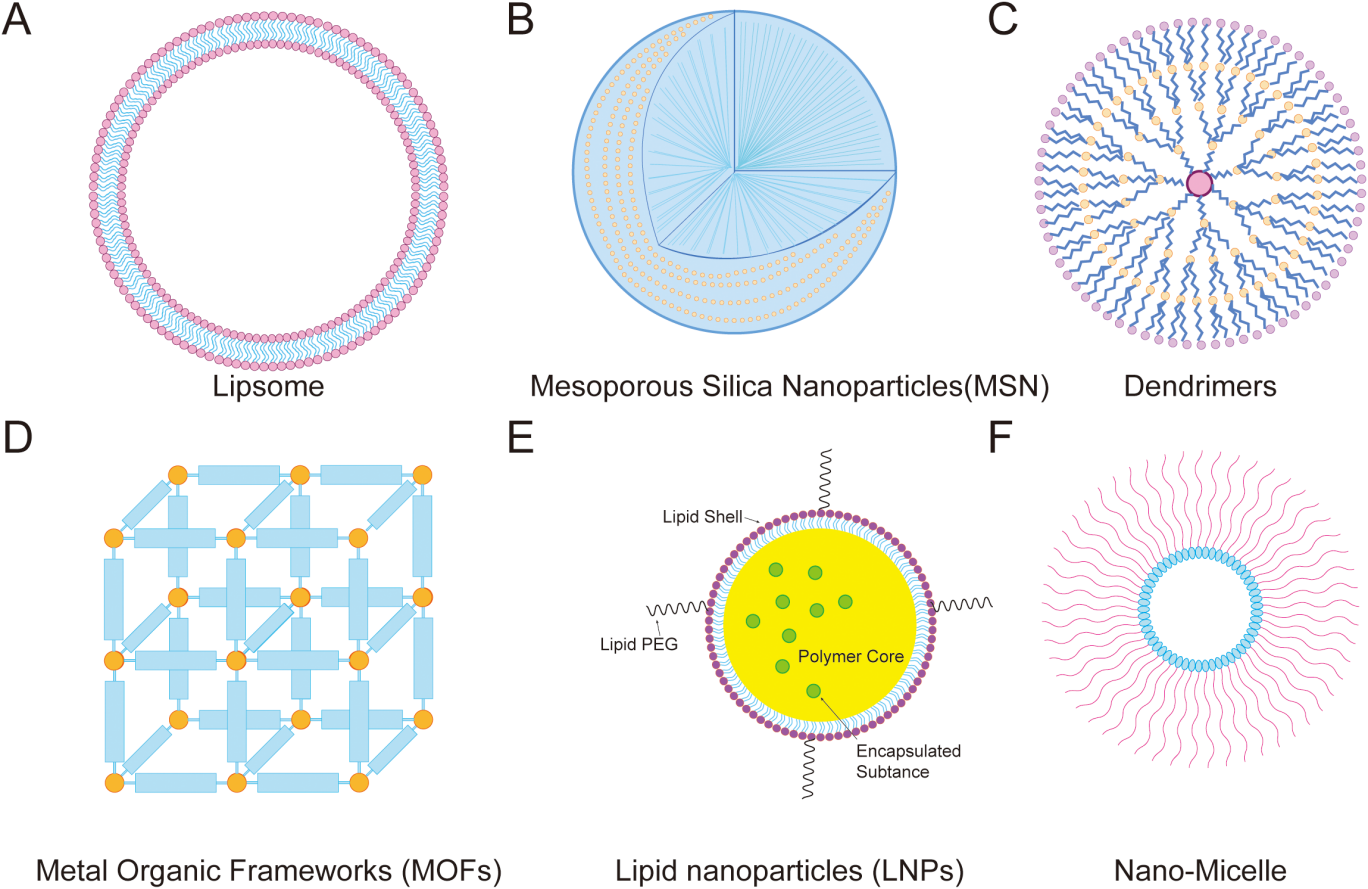
Mitochondrial-targeted nanodrug delivery strategies. **(A)** Lipsome, **(B)** Mesoporous Silica Nanoparticles(MSN), **(C)** Dendrimers, **(D)** Metal Organic Frameworks (MOFs), **(E)** Lipid nanoparticles (LNPs), and **(F)** Nano-Micelle.

Anti-tumor drugs can be loaded into various carriers such as liposomes, polymer nanoparticles, micelles, and solid lipid nanoparticles to work in conjunction with mitochondria-targeted modifications to achieve better anti-tumor effects (186) (**Figure 8**).

#### 3.3.1 Mitochondria-targeted liposomes

Liposomes are artificial membranes with bilayers, which are generally prepared by high-pressure homogenization, ethanol injection, rotary evaporation and ultrasound, and microfluidics. They can carry hydrophilic and lipophilic drugs, with the former distributed in the core compartment and the latter distributed in the bilayer membrane (187). Liposomes have attracted extensive attention due to their excellent drug delivery capabilities, biocompatibility, biodegradability, and ease of manufacture. Mitochondria-targeted liposomes have advantages in tumor-targeted therapies (188).

Liposomes enhance mitochondrial uptake of DOX and the chemosensitizer lonidamine (LND) by cancer cells, inhibiting tumor cell proliferation and inducing cell apoptosis. Lip-SPG significantly alters mitochondrial functions including reduced production of intracellular ATP, induction of ROS production, and enhancing ΔΨm depolarization (189). Milpoxetine (MPt)-loaded liposomes target mitochondria and trigger mtDNA replication blockage to induce mitophagy (190). DOX-loaded liposomes localize to mitochondria, and generate higher ROS levels (191).

Mitochondria-targeted photosensitizer liposomes exhibit high photodynamic therapy efficiency. Nanophotosensitizers can monitor abnormal mitochondrial morphology during photodynamic therapy under the guidance of fluorescence imaging. Liposome-encapsulated photosensitizers enhance cellular uptake, localize in mitochondria, and enhance anti-angiogenesis in PDT treatment (192). After TPP-modified liposomes are internalized by cells, a large amount of ROS can be generated upon laser irradiation, and a stimulatory effect on STING activation and enhanced infiltration of anti-tumor immune cells is observed, which can be used for PDT treatment (193).

#### 3.3.2 Mitochondria-targeted mesoporous silica nanoparticles

MSNs are an ordered mesoporous material prepared by sol-gel methodologies including microwave-assisted technology, chemical etching technology, and template methods. The material has characteristics of good biocompatibility, high specific surface area, controllable size, and degradability. MSN can improve the targeting of drugs in tumor mitochondria through direct coupling with drugs and enhance the killing effect of tumors. MSNs can efficiently deliver DOX and α-TOS to tumor cell mitochondria, enhancing cancer cell killing effects (194, 195). MSN modified Bcl-2 conversion peptides enter mitochondria and bind to Bcl-2, exposing the BH3 domain and inducing apoptosis of DOX-resistant cells (196). MSN increases the accumulation of folate membrane cell receptors (folate) in tumor cells and targets mitochondria (197). MicroRNA-31 coupled to MSNs loaded with DOX increases active transport and promotes intracellular accumulation of drugs. MicroRNA-31 not only directs targeted mtEF4 to promote cell death, but also has a synergistic effect when used in combination with DOX (198). Phenylboronic acid (PBA)-labeled MSN carriers induce mitochondria-dependent apoptosis in MCF-7 cells through oxidative stress (199).

MSNs can also be combined with mitochondria targeting strategies such as DLC or MPP to further improve the targeting effect of drugs in tumor mitochondria. Pt-loaded MSNs achieve ROS burst in mitochondria, leading to cell apoptosis (200). Mesoporous connections of MSNs can deliver DOX to mitochondria and enhance copper consumption by producing H_2_O_2_ (201). After blocking surface pores through disulfide bonds, MSNs can target cancer cells with DOX, penetrate the cell membrane and quickly release anticancer drugs and mitochondria-targeted peptides, and induce significant synergistic anticancer effects (202).

MSNs can target the delivery of photosensitive and thermosensitive drugs to mitochondria, increase ROS, and enhance the efficacy and tumor imaging of new treatments such as tumor PDT. α-Tocopherol succinate and indocyanine green (IDG) MSNs reduce innate oxygen consumption by blocking the mitochondrial respiratory chain, leading to endogenous mitochondrial ROS burst and imaging-guided PDT (203). IR780-loaded MSNs nanoparticles can accumulate in tumors, destroy mitochondria and inhibit cellular respiration by decomposing H_2_O_2_, resulting in sustained reduction of hypoxia in tumor tissues, thereby enhancing the therapeutic effect of PDT (204). Redox-responsive drugs delivered by MSNs target mitochondria in living cells and induce apoptosis derived from mitochondrial membrane depolarization (205).

#### 3.3.3 Mitochondria-targeted dendrimers

Dendrimers have a hyperbranched structure that can fill hydrophobic drug small molecules into polymer gaps and graft drugs onto polymer chains. Targeted dendrimer curcumin (TDC) colocalizes with mitochondria of cancer cells, inducing potent apoptosis and cell cycle arrest. It reduces ATP and glutathione and increases ROS levels in isolated mitochondria of rat hepatocytes (206). Poly(amidoamine) (PAMAM) is a common dendrimer targeting strategy with the ability to effectively regulate dendrimer targeting mitochondria. Active targeting of dendrimers induces P-glycoprotein (P-gp) overexpression and apoptosis in multidrug-resistant cells (207). TPP conjugated to PAMAM dendrimers, optimizes the density of surface TPP by adjusting the length of TPP-PEG linker, enhancing mitochondrial targeting ability and antitumor bioactivity (208).

#### 3.3.4 Mitochondrial-targeted metal-organic frameworks

Metal-organic frameworks (MOFs) are a class of porous materials formed by the coordination of inorganic metal ions and organic ligands. Compared with other nano-drug carriers, MOFs have the advantages of high porosity, adjustable structure, controllable size, and easy modification. In addition, MOFs exhibit unique advantages: (1) easy preparation and good stability, assembled from non-toxic metals (Fe, Zn, Ca, Mg, etc.) and low-toxic carboxylic acids or phosphonic acids; (2) biodegradable, especially when exposed to water; (3) an internal microenvironment suitable for the delivery of drug molecules with different activities (209). These properties make MOFs ideal materials for biomedical applications, such as the delivery of drugs or imaging agents. Surface modification of materials further enriches the approach of using MOF as a drug delivery platform to treat diseases, such as PTT combined with chemotherapy, ultrasound therapy combined with chemotherapy, and other combination treatment strategies (210).

MOFs encapsulated in macrophage-cancer hybrid membranes (MCHMs) enhance the cancer homing targeting ability of nanoparticles (NPs), damage ΔΨm, and lead to cancer cell apoptosis (211). The ZCProP nanoplatform triggers cell ferroptosis through cuproposis and inhibits the anti-ferroptosis protein glutathione peroxidase 4 (212). MOFs can deliver oxymatrine (Om) and astragaloside IV (As) into the HCC microenvironment, and increase the oxygen consumption rate and proton efflux rate of tumor-infiltrating lymphocytes (TILs) by regulating the mitochondrial function of CAFs and TILs (213). The mitochondrial targeting drug of gallium-based organic frameworks produces ROS and releases L-Arg, which reacts with ROS to generate NO, downregulates HIF-1α expression to improve tumor hypoxia, and enhances immune responses by increasing calreticulin (CRT), high-mobility group box 1 (HMGB1), and T cell proliferation (214). The metal-organic framework GCZMT targets mitochondria and releases NO under MW irradiation, interfering with the cell's energy supply and inhibiting tumor cell growth. Upregulation of heat shock protein (HSP)70 expression can facilitate CD4^+^ and CD8^+^ T cell activation to promote anti-tumor immunity (215).

#### 3.3.5 Other mitochondria-targeted nanoparticles

Lipid-polymer nanoparticles (LPNPs) are composed of a polymer core and a biocompatible lipid shell. LPNPs have a longer half-life compared with conventional liposomes. After lipid-polymer hybrid nanoparticles are taken up by cancer cells, the surface charge of LPNPs is restored due to the separation of PEG under intracellular reducing conditions, resulting in rapid and precise targeting of mitochondria (216). Nanomicelles are nanocarriers with a core-shell structure formed by self-assembly of amphiphilic copolymers in aqueous media, which have the advantages of simple preparation and small particle size. Micelles not only significantly improve drug solubility, but also increase drug accumulation in tumor sites through enhanced permeability and retention (EPR), and they improve the effect of chemotherapy and can partially reverse tumor drug resistance (217). Mitochondria-targeted polymeric micelles (OPDEA-PDCA) target mitochondria and induce mitochondrial oxidative stress via inhibition of pyruvate dehydrogenase kinase 1(PDHK1), leading to immunogenic pyroptosis in osteosarcoma cell lines (218). Charge-reversible nanocopolymers are copolymers that are modified with anions to shield the positive charge of the nanosystem, avoiding nonspecific binding with other proteins and subsequent elimination (219). In normal physiological environments (pH 7.4), the copolymers are neutral or negatively charged, which can reduce the uptake of nanomedicines by macrophages in the reticuloendothelial system while ensuring their stability in the blood circulation. However, when they reach the tumor site, their potential undergoes a charge reversal, and their affinity with the tumor cell surface is significantly enhanced, leading to the accumulation of nanoparticles in tumor cells (220). Tumor acidity triggers charge reversal and mitochondrial targeting activation of TPP-containing nanomedicines, which is a simple and effective strategy for delivering DOX to cancer cell mitochondria and overcoming DOX resistance in MCF-7/ADR breast tumor cells *in vitro* and *in vivo (* 120).

In summary, mitochondrial-targeted drug delivery systems can deliver drugs to tumor sites and enhance the effectiveness of cancer treatment through various mechanisms, including disrupting mitochondrial energy metabolism, regulating ROS levels, modulating cell death-related proteins, damaging mitochondrial DNA, and regulating mitophagy. Liposomes can carry both hydrophilic and lipophilic drugs, enhancing drug uptake and inducing mitophagy, while their photosensitizer liposomes can also assist in photodynamic therapy (PDT). MSN boasts good biocompatibility and high surface area, allowing for direct drug conjugation or the integration of other targeted strategies, thus enhancing drug targeting and cytotoxic effects, and can also deliver photothermal sensitizers. Dendritic polymers are suitable for carrying hydrophobic drugs. MOFs are porous, structurally tunable, and biodegradable, making them apt for delivering drugs or imaging agents, and they can also be employed in combination therapies. Additionally, LPNP has a long half-life, nanomicelles can reverse drug resistance, and charge-reversible nanocopolymers offer precise targeting. These systems can improve cancer treatment outcomes through various approaches, making them a promising therapeutic strategy, reducing the high risks associated with traditional treatments such as surgery.

## 4 Mechanism of action of mitochondria-targeted therapy strategies in tumor immunity

Mitochondrial dysfunction plays an important role in tumor-induced immunosuppression. Mitochondrial-targeted drugs that restore mitochondrial function may overcome this dilemma and improve the efficacy of cancer therapies (221). Mechanisms of action for mitochondrial-targeted drugs in enhancing tumor immunity include targeting of mitochondrial metabolism, mitochondrial ROS, ICD, mtDNA and immune checkpoints.

### 4.1 Targeting mitochondrial metabolic pathways to improve tumor immunotherapy

#### 4.1.1 Targeting glycolysis metabolic pathways

**Figure 9:**
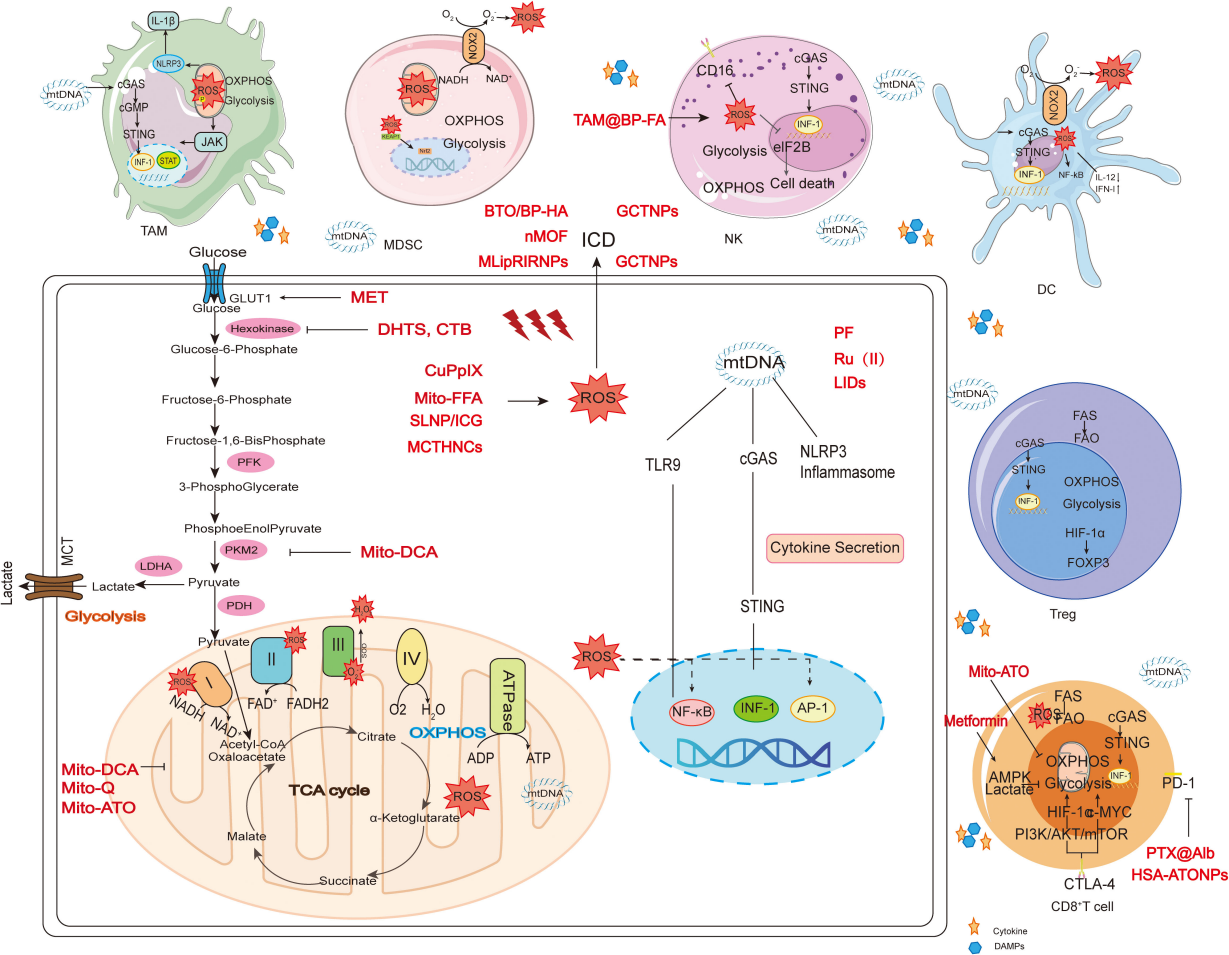
Employing mitochondria-targeted drugs to alter immune regulation. Mitochondrial targeting strategy drugs can affect tumor immunity through multiple pathways. Acting on glycolysis such as MET, DHTS, Mito-DCA; Acting on ROS such as CuPpIX, Mito-FFA, and SLNP/ICG; Acting on OXPHOS: Mito-DCA, Mito-Q, Mito-ATO; Acting on ICD such as nMOF, MLipRIRNPs, BTO/BP-HA; Acting on mtDNA such as PF, Ru (II), and LIDs; Acting on CD8+ T cell glucose metabolism such as metformin and Mito-ATO; Acting on PD-1 such as PTX@Alb and HSA-ATONPs.

Glucose is one of the main players in tumor progression and a promoter of tumor invasion and metastasis. Glycolysis rapidly produces ATP, which provides sufficient energy for tumor cell proliferation. Under aerobic or hypoxic conditions, tumor cells have enhanced glycolytic activity and reduced mitochondrial respiration. Therefore, reversing the high glycolytic state of tumor cells to induce cell death is a possible approach to cancer treatment. Key glycolytic genes include glucose transporter 1 (GLUT1), hexokinase 2 (HK2), pyruvate kinase-M2 splicing isoform (PKM2) and lactate dehydrogenase (LDH-A) (**Figure 9**).

Glucose uptake provides a key metabolic control point by targeting the Glut family of glucose transporters (120). Interference with HK1/2 and GLUT1 function in hematopoietic cells inhibits glycolysis, reduces ATP production, enhances the apoptotic effect, and preserves normal CD34^+^ bone marrow progenitors (222). Glycosylated poly(amidoamine)/celastrol (PAMAM/Cel) complexes are characterized by high photothermal conversion efficiency, hypoxia-sensitive PEG outer layer detachment, and alkali-sensitive drug release. The complexes show specific cellular uptake and accumulation in tumor cell mitochondria in hypoxic environments that overexpress GLUT1 (223). Met administration elevates mtROS and cell surface Glut-1, leading to IFN-γ production in CD8^+^ TILs in tumor cells (224).

Hexokinase-2 (HK2) is located at mitochondrial-endoplasmic reticulum (ER) contact sites called mitochondrial associated membranes (MAMs). HK2 expression is significantly elevated in most HCC cell lines and tumor tissues compared to normal cell lines and tissues (225). Since HK2 is the major rate-limiting enzyme in the aerobic glycolysis pathway, inhibiting HK2 is an effective strategy to block glycolysis. HK2 degraders cause mitochondrial damage and then induce GSDME-dependent pyroptosis and ICD, resulting in increased anti-tumor immunity (226). HK2-targeting peptides trigger mitochondrial Ca2^+^ overload, leading to Ca2^+^-dependent calpain activation, mitochondrial depolarization and cell death, and can also cause massive death of chronic lymphocytic leukemia B cells (227). Dihydrotanshinone I (DHTS) reverses metabolic reprogramming in colon cancer by inhibiting hexokinase activity and free fatty acids (FFA) via the PTEN/AKT/HIF1α-mediated signaling pathway (228).

Pyruvate kinase (PK) is a key enzyme that regulates the last step of glycolysis, catalyzing phosphoenolpyruvate (PEP) and ADP to generate pyruvate and ATP. Pyruvate kinase includes erythrocyte/liver pyruvate kinase (PKLR) and muscle pyruvate kinase (PKM), and PKM has two isoforms, PKM1 and PKM2. Mitochondra-targeted dichloroacetate (Mito-DCA) enhances delivery of DCA to mitochondria, leading to a shift from glycolysis to glucose oxidation, cell death through apoptosis, and increses DCs secretion of IL-12 (229). ATO treatment inhibits oxygen consumption and metabolically induces aerobic glycolysis and oxidative stress, and also induces apoptosis in CD44^+^/CD24^low/-^ and ALDH^+^ cancer stem cells (230).

Another way to target glycolysis is to reduce levels of ATP. The mechanism of Met's anti-tumor effect may be that it directly reduces the production of ATP due to its function as an inhibitor of mitochondrial electron transfer chain complex I and an activator of AMPK (132). Metformin (Met) reduces tumor-infiltrating Treg (Ti-Treg), especially in the terminally differentiated CD103^+^KLRG1^+^ population, and also reduces expression of immune suppressive effector molecules such as CTLA4 and IL-10. Met inhibits the differentiation of naive CD4^+^ T cells into induced Treg (iTreg) by reducing forkhead box P3 (Foxp3) protein expression, which is determined by the increase of phosphorylated S6 (pS6), a downstream molecule of mTORC1. Rapamycin and compound C (AMPK inhibitor) restores the generation of iTreg, further indicating that mTORC1 and AMPK are involved (231).

A variety of strategies targeting key glycolytic genes (such as GLUT1, HK2, PK, etc.) can induce tumor cell death or enhance anti-tumor immunity by reducing ATP production and triggering mitochondrial damage. By inhibiting the mitochondrial electron transfer chain complex and activating AMPK, ATP production is directly reduced, which affects tumor immune cells and plays an anti-tumor role.

#### 4.1.2 Targeting mitochondrial OXPHOS

The "Warburg effect" is necessary for tumor cells to undergo malignant transformation and escape immune attack. Reversing the Warburg effect by using drugs to intervene in the metabolic behavior of tumor cells so that their main energy production pathway changes from glycolysis to OXPHOS, may be a promising therapeutic strategy for treating such tumors. Upregulating the expression of genes related to mitochondrial biogenesis, thereby increasing mitochondrial activity and reprogramming cellular energy metabolism, produces an "anti-Warburg effect", and is known to regulate the differentiation of glioblastoma cells towards normal cell phenotype (118). Mitochondria-targeted OXPHOS inhibitors reduce hypoxia in tumor cells in a dose-dependent manner, potentially sensitizing hypoxic tumor cells to radiotherapy (232). Mitoquinone (Mito-Q) adsorbed to the inner mitochondrial membrane blocks ATP synthase, dissipates ΔΨm in HepG2 cells, and induces uncoupling of autophagy from OXPHOS in cancer cells (233). Functionalized Ir(III) complexes selectively localize in mitochondria and generate singlet oxygen and superoxide anion radicals upon two-photon irradiation, leading to the disruption of the mitochondrial respiratory chain, thereby interfering with mitochondrial OXPHOS and glycolytic metabolism, triggering cell death by combining ICD and ferritin autophagy (234).

Metabolic reprogramming of T cells in the TME impairs effector T cell responses against tumor cells. Mitochondrial-targeted drugs modulate the immune microenvironment via OXPHOS. Mitochondria-targeted hydroxyurea (Mito-HU) reduces mitochondrial complex I and complex III-induced oxygen consumption, effectively inhibits monocytic MDSCs and suppressive neutrophils, and stimulates T cell responses (235). Mito-CI reduces mitochondrial complex I oxygen consumption and Akt-FOXO signaling, blocks cell cycle progression, melanoma cell proliferation, and inhibits tumor progression. Anti-proliferative properties of mitochondria-targeted complex I inhibitors (Mito-CI) inhibit differentiation, viability, and suppressive function of bone marrow-derived MDSCs and increase activation of T cells (236). Mitochondria-targeted ATO inhibits the expression of mitochondrial complex components, OXPHOS, and glycolysis genes in granulocytic-MDSCs and Treg (237). The resulting reduction in intra-tumoral granulocytic-MDSCs (G-MDSCs) and Treg could contribute to the observed increase in tumor-infiltrating CD4^+^ T cells. In contrast, Mito-ATO significantly inhibits OXPHOS and glycolysis in G-MDSCs. These observations support the predicted higher OXPHOS and glycolysis in effector memory CD8^+^ T cells and lower OXPHOS and glycolysis in G-MDSCs after Mito-ATO treatment (238). AMPK is considered a major intracellular energy sensor and key regulator of mitochondrial biogenesis that can control metabolic reprogramming in immune cells and enhance anti-tumor immunity. The AMPK activator Met is considered a candidate drug to improve cancer treatment efficacy by interfering with tumor metabolic reprogramming (239). Met increases the numbers of CD8^+^ T cells and protects them from apoptosis and exhaustion (240).

Mitochondria-targeted OXPHOS inhibitors can induce tumor cell death or enhance their sensitivity to radiotherapy by affecting mitochondrial function and interfering with energy metabolism. Mitochondria-targeted drugs can also improve TME, stimulate T cell responses, protect CD8^+^ T cells and enhance anti-tumor immunity by regulating OXPHOS.

### 4.2 Targeting mtROS to improve tumor immunotherapy

Since ROS in tumor cell mitochondria have been shown to play an important role in immunotherapy, how mitochondrial targeted drugs can promote tumor cells to produce excessive ROS, damage mtDNA, release immunogenic intracellular substrates, and activate anti-tumor immunity are important areas of focus in anti-tumor research (**Figure 9**). Cu-modified protoporphyrin(CuPpIX) can be delivered to the mitochondria, inducing ROS burst *in situ* under ultrasound irradiation, which leads to severe mitochondrial dysfunction and amplies ICD to stimulate the body's immune response and enhance the infiltration of CD8^+^ T cells into tumors (241). The nanosystem delivers Ca@GOx to mitochondria, inducing mitochondrial Ca2^+^ overload and generating high levels of ROS, leading to pyroptosis and promoting tumor infiltration of CD8^+^ T cells (242). Indocyanine green (ICG) nanoparticles (SLNP/ICG) stimulate glioma cells to produce abundant ROS under NIR irradiation, activate mitochondria-mediated apoptosis pathways, and increase CD4^+^ and CD8^+^ T cell proliferation (243). The PDT photosensitizer IR700DX generates ROS upon light irradiation and promotes downstream p38 phosphorylation and CASP3-mediated gasderminE (GSDME) cleavage, which induces pyroptosis, triggers ICD, and enhances the anti-cancer efficacy of PD-1 blockade (244). The nanoplatform CS@KET/P780NPs induces apoptosis by enhancing ROS accumulation, which triggers ICD and a long-term antitumor response by releasing tumor-associated antigens (TAA) and DAMPs (245). The molecular photosensitizer FEPT, used in NIR-II, generates ROS and hyperthermia under laser irradiation, leading to mitochondrial dysfunction and light-induced apoptosis through the caspase-3 pathway, releasing immunogenic intracellular substrates, and thus promotes activation of anti-tumor immunity (246). Photosensitizer IR780 triggers ROS production through a Fenton-like reaction, induces ferroptosis of tumor cells, and simultaneously induces DC maturation, promotes cytotoxic T lymphocyte infiltration, decreases immune suppression in the tumor microenvironment, and activates a systemic immune response (247). Sonodynamic therapy (SDT) drugs stimulate ROS production and reduce ΔΨm, and induce antitumor immune responses by upregulating NK cell activity and reducing numbers of immunosuppressive macrophages (248). Mitochondria-targeted FFa (Mito-FFa) increases the production of mtROS that triggers endoplasmic reticulum (ER) stress, and also oxidizes mtDNA and promotes its leakage into the cytoplasm. This leads to cGAS-STING-dependent IFN-I secretion, improving tumor antigen uptake, DC maturation, and cross-priming of CD8^+^ T cells (249). Mitochondria-targeted liposomes induce massive lipid peroxidation and increase ROS in mitochondria, ultimately triggering ferroptosis in bladder cancer cells, promoting the release of intracellular DAMPs, inducing the release of mtDNA into the cytoplasm, activating the cGAS-STING pathway to secrete IFN-β, and increasing DC cross-presentation of antigens to T cells, and increasing macrophage phagocytosis (250).

Mitochondria-targeted drugs can activate the cGAS-STING pathway, promote DAMPs release, induce ferroptosis, and regulate immune cell activity by producing MTROS, reducing δψm, triggering endoplasmic reticulum stress or Lipid peroxidation, etc. The reduction of immunosuppressive cells will ultimately improve the efficacy of tumor immunotherapy.

### 4.3 Targeted mitochondrial-mediated ICD

ICD is a form of cell death that releases TAA and tumor-specific antigens (TSA), and provides "danger signals" to facilitate generation of an effective T cell response. It is characterized by the release and/or increased expression of DAMPs, precursor antigens, inflammatory cytokines, and inflammatory mediators. The main DAMPs include ATP, calreticulin (CRT), high mobility group box protein B1 (HMGB1), heat shock protein (HSP), type I interferon (IFN I) and Annexin 1 (ANXA1), which then activate and recruit APCs such as macrophages and DCs to activate T cells reactive to tumor antigens. DAMPs bind to pattern recognition receptors (PRRs) to induce a series of immune events (251). Mitochondria-targeted drugs trigger ICD by releasing ROS. Mitochondrial-targeting liposomal nanoparticles cause severe ferroptosis of tumor cells and trigger ICD through the accumulation of lipid peroxides, activating anti-tumor immunity (252).

The released DAMPs promote DC maturation, activate cytotoxic T lymphocytes, and reverse immune suppression in the tumor microenvironment. Polymeric nanoparticles induce mitochondrial dysfunction and amplify endoplasmic reticulum stress, leading to tumor cell apoptosis and ICD, which promotes DC maturation and increases numbers of tumor-infiltrating cytotoxic T lymphocytes (253). DOX-containing liposomes induce effective ICD in targeted cancer cells to promote DC maturation and stimulate T cell proliferation and activation, transforming the immunosuppressive tumor microenvironment (ITM) into an immune responsive environment (254). Toll-like receptor agonist R837 synergistically promotes DC maturation. Promoting DC activation by inducing ICDs resulted in more robust antitumor efficacy, which can inhibit metastatic disease progression and promote the development of durable antitumor memory responses (255).

DAMPs released by ICD effectively induce M1 polarization and migration of the polarized macrophages. TPP-modified CuET triggers ICD, and the released DAMPs induce macrophage M1 polarization and migration to activate an immune response consisting of CD8^+^ T cells and NK cells (256). SDT promotes the maturation of DCs and increases numbers of infiltrating immune cells. M2 macrophages are also re-polarized to an M1 phenotype and MDSCs are depleted to reverse immunosuppression and enhance immune responses (257).

Mitochondria-targeting drugs can trigger ICD by releasing ROS and other DAMPs, which can promote DC maturation, activate cytotoxic T lymphocyte, and reverse immunosuppression in the tumor microenvironment. They can also induce macrophage M1 polarization and migration, and SDT can also enhance the immune response by regulating the function of ICD-associated immune cells (such as macrophages, MDSCs).

### 4.4 Targeting mitochondria autophagy to increase tumor immunity

Mitochondrial autophagy, as a core mechanism regulating mitochondrial homeostasis, exerts bidirectional effects in tumor immunity by influencing immune cell function, tumor microenvironment metabolism, and innate immune pathways: It can maintain effector function by clearing damaged mitochondria from immune cells and enhance immune responses by activating the STING pathway. It also promotes antitumor effects by facilitating antigen presentation and activating effector immune cells. Conversely, it can promote immune escape by degrading MHC molecules, supporting immune-suppressive cells, and regulating immune checkpoints.

The autophagy inhibitor chloroquine (CQ) induces Ca²^+^ release through lysosomal Ca²^+^ channels, activating p38 and NF-κB to reprogram TAMs from M2 to M1 phenotypes, eliminating cancer cell resistance and achieving enhanced therapeutic effects (258). The autophagy inhibitor bafamilomycin confers ADCC resistance by altering cell death, modulating immunoregulatory factors in NK and/or cancer cells, and regulating HER2 kinetics (259). Spongy calcium carbonate (CaCO_3_) nanoparticles disrupt DC autophagy and antigen cross-presentation, enhancing DAMP release from tumor cells to improve DC maturation (260).

Autophagy activator like rapamycin enhances NK cell cytotoxicity by upregulating IL-27R expression, thereby restricting tumor growth (261). Metformin significantly activates AMPK signaling, reducing Th1 and Th17 cells while increasing Th2 and Treg cells (262). Combined with nelfinavir, it induces SIRT3/mROS-dependent autophagy and sensitizes NK cells against human cervical cancer cells (262). Temsirolimus (TEM) activates autophagy to suppress tumor-derived sEV PD-L1 secretion and increase the number and activation of CD4^+^and CD8^+^T cells, inducing systemic anticancer immunity (263). PD-1 blockade combined with endostar significantly suppressed tumor growth, leading to reduced IL-17 and TGF-β1 levels, increased IFN-γ secretion, decreased MDSC, and reversal of CD8+ T cell suppression. It improved the tumor microenvironment and activated autophagy (264). The nanomaterial MoO_3_-x nanowires (MoO_3_-x NWs) combined with PTT activated autophagy, induced DC maturation and antigen presentation, subsequently activating CD8^+^ T cell-mediated adaptive immunity. It promoted TAM polarization toward M1 macrophages, suppressed Treg cell infiltration at tumor sites, and alleviated immune suppression in the tumor microenvironment (265). Lanthanum nickel oxide (LNO) nanoenzyme-induced macrophage autophagy promotes macrophage M1 polarization (266).

Natural compounds exhibit bidirectional effects on autophagy, further influencing tumor immunity. (-)-Guaiol inhibits tumor growth by inducing autophagy, inducing ICD, enhancing DC activation, and boosting T cell infiltration (267). Berberine hydrochloride (Ber) increases autophagosome accumulation, elevates LC3-II and p62 levels in melanoma cells to enhance MHC-I-mediated antigen presentation, and improves CD8+ T cell infiltration (268). Rocaglamide (RocA) targets ULK1 to inhibit autophagy and restore NK cell GZMB levels, activating cGAS-STING signaling to promote NK cell infiltration (269). Naringenin can at least partially induce BC cell inhibition of autophagy and cell proliferation by modulating the FKBP4/NR3C1/NRF2 signaling pathway, while also enhancing DC differentiation and maturation (270).

### 4.5 Targeting immune checkpoints to increase tumor immunity

Immune checkpoints are a class of immune inhibitory molecules that are expressed on immune cells to regulate the degree of immune activation. These molecules are known to play an important role in preventing the occurrence of autoimmunity, but they can also be hijacked by cancers to inhibit tumor immune responses. Mitochondria-targeted treatments can be used in conjunction with immune checkpoint therapies to facilitate better induction of tumor-reactive T cells. The mitochondria-targeted drug Met attenuates upregulation of the immune checkpoints programmed cell death protein 1 (PD-1) and lymphocyte activation gene-3 (LAG-3), thereby increasing CD8^+^ T cell infiltration and survival in the harsh tumor microenvironment (271). Synergistic effects between ATO and anti-PD-L1, which blocks binding of PD-L1 to its receptor PD-1, leads to the activation of tumor-reactive CD8^+^ T cells and promotes the establishment of tumor-specific immune memory (272).

Mitochondria-targeted drugs can reverse tumor hypoxia, inhibit PD-L1 expression, thereby enhancing therapeutic effects. Albumin-bound paclitaxel (PTX@Alb) accumulates in 4T1 breast tumors and reduces the expression of PD-L1 and TGF-β, resulting in enhanced T cell infiltration of the tumors (273). Mitochondria-targeted ATO promotes CD8^+^ T cell recruitment by reducing tumor hypoxia, and ATO treatment enhances the efficacy of anti-PD-1 immunotherapy (274). MHI-TMX@ALB nanoparticle photosensitizers can reverse tumor hypoxia and inhibit PD-L1 protein expression in the tumor microenvironment, resulting in enhanced efficacy of photodynamic immunotherapy by increasing T cell infiltration (275).

Mitochondria-targeted drugs induce ICD, reverse the immunosuppressive TME, and promote immune checkpoint blockade therapy. MOF induces ICD after ultrasound exposure to promote DC activation. It can achieve *in vivo* synergy with anti-CTLA-4 immune checkpoint blockade to reverse the ITM (255). Nanodiagnostic therapeutics stimulate ICD, leading to the massive release of TAA and DAMPs, thereby improving the ITM and providing another treatment that can be combined with immune checkpoint blockade therapy (276). SDT leads to local production of ROS after ultrasound irradiation, damaging tumor cell mitochondria, downregulating PD-L1 expression, and promoting ICD (277). Zero-valent-iron nanoparticles (ZVI-NP) enhanced anti-tumor immunity by re-polarizing pro-tumor M2 macrophages to anti-tumor M1 macrophages, reducing the number of Treg, downregulating PD-1 and CTLA4 on CD8^+^ T cells to enhance their cytolytic activity against cancer cells, and reducing expression of PD-L1 on the tumor (278).

Mitochondrial targeted therapy can be combined with immune checkpoint therapy to synergistically activate tumor-reactive CD8^+^ T cells and establish immune memory. Mitochondria-targeted drugs can also synergize with immune checkpoint blockade therapy by reversing tumor hypoxia, inhibiting PD-L1 expression, or inducing ICD, reversing immunosuppression TME, and reducing tumor necrosis factor-α expression, enhance the effect of photodynamic immunotherapy, enhance T cell lytic activity, and then enhance tumor immunity.

### 4.6 Improving chimeric antigen receptor T cell therapy

Chimeric antigen receptor T (CAR-T) cell therapy is an exciting form of immunotherapy that is being used to primarily treat patients with hematologic cancers (leukemia, lymphoma, multiple myeloma), but also holds promise for treating solid tumors. CARs are constructed through genetic technology, where a gene encoding the CAR that recognizes antigen(s) on tumor cells is introduced into T cells. Upon CAR recognition of cognate antigen(s) on tumor cells, intracellular co-signaling domains in the CAR trigger T cell activation and killing of the tumor cells. Essentially, the CAR adds a new antigen binding receptor on the surface of T cells that is independent of MHC/antigen presentation. Despite impressive early clinical responses after CAR-T cell therapy, many patients still experience disease relapse, which is frequently due to loss of target antigen(s) on the tumor cells, so improvements to this form of cancer immunotherapy are warranted.

Increased mitochondrial biomass preserves bioenergetic potential to meet the metabolic demands of activated T cells. When T cells are modified to generate CAR-T cells, a unique mitochondrial adaptation is required to establish stemness and persistence of the CAR-T cells (279). Targeting mitochondrial metabolism to promote T cell memory formation and metabolic adaptation may represent an attractive strategy to improve CAR-T cell therapies and other immunotherapies (280).

CD8^+^ T cell migration depends on mitochondrial oxidation of glucose and glutamine and both ATP and ROS production. Drug interventions that increase mitochondrial activity can improve CAR-T cell recruitment to tumors, thereby better controlling tumor growth (281). Knockout of endogenous TCR in ARI-0001 CAR-T cells increases the percentage of energetic mitochondria (282). Nuclear receptor 4A (NR4A)1/2/3 triple knockout CAR-T cells show enhanced mitochondrial OXPHOS, increasing the persistence and stemness of CAR-T cells (283). Addition of the cosignaling molecule 4-1BB to the CAR construct promotes memory T cell respiratory capacity, increases fatty acid oxidation and enhances mitochondrial biogenesis, generating increased numbers of T cells with an effector-memory cell phenotype (284).

Upregulating T cell mitochondrial plasticity to increase the efficacy of adoptive cellular immunotherapies (ACI), including CAR-T cells, will generate T cells with strong metabolic adaptability and durable immune function, thereby preventing tumor metastasis and recurrence (285). Targeting methylation-controlled J protein (MCJ) in CD8^+^ CAR-T cells can increase mitochondrial metabolism and improve the anti-tumor activity of CAR-T therapy (286). CAR-T cells prevent staurosporine (STS)-induced apoptosis of human CD3^+^ T cells by interfering with the caspase pathway and improving their metabolic fitness and resistance to environmental stress (287).

Mitochondria are essential for CAR-T cells, and strategies targeting mitochondrial metabolism, such as increasing mitochondrial biomass, enhancing mitochondrial activity with OXPHOS, upregulating mitochondrial plasticity, and so on, have been proposed to reduce mitochondrial plasticity in CAR-T cells. They can improve the stemness, persistence, metabolic adaptability and anti-tumor activity of CAR-T cells, and help to improve the therapeutic effect.

### 4.7 Targeting mtDNA to improve tumor immunotherapy

Since tumor cell mitochondria are typically dysfunctional due to mutations in mtDNA, correcting mtDNA mutations is considered an effective strategy to restore mitochondrial function. Mitochondria-targeted drugs induce tumor mtDNA oxidation and specific release into the cytoplasm, activating the cGAS-STING pathway and affecting tumor immune-related responses. Mitochondrial lipid peroxidation and ROS promote the release of intracellular DAMPs, thereby facilitating the release of mtDNA into the cytoplasm, which activates the cGAS-STING pathway and increases cross-presentation of antigens by DCs and macrophages. Ultimately, this process induces CD8^+^ T cell infiltration into the TME to inhibit tumor growth (288).

Under NIR irradiation, mitochondria-targeted ROS are generated and mtDNA released to provide endogenous danger-associated molecules that activate the cGAS-STING pathway, promoting the maturation of DCs and the infiltration of cytotoxic T lymphocytes (289). Treatment with mitochondria-targeting gold(I) complexes generates large amounts of ROS and promotes DNA excretion. The ROS induces ICD and the released DNA activates the cGAS-STING pathway, generating a strong anti-cancer immune response (290). Indocyanine green and doxorubicin plus ultrasound enhances the nuclear delivery of doxorubicin, induces tumor mitochondrial DNA oxidation, activates cGAS-STING signaling, and triggers anti-tumor T cell immunity (291).

Mitochondrion-targeting drugs can induce tumor mtDNA oxidation and release to the cytoplasm, activate the cGAS-STING pathway, while mitochondrion Lipid peroxidation and ROS can also promote mtDNA release. This in turn promotes antigen cross-presentation, DC maturation, and CD8^+^ T cell infiltration to trigger anti-tumor immune responses.

In summary, mitochondrial targeting offers various strategies to enhance tumor immunotherapy. Targeting metabolic pathways can regulate immune cell function by inhibiting key enzymes in glycolysis (such as GLUT1 and HK2) or modulating OXPHOS to reverse the Warburg effect; targeting mtROS can induce excessive mitochondrial ROS, damage mitochondrial DNA, trigger immunogenic cell death (ICD), and activate anti-tumor immunity. Targeting mitochondria-mediated ICD can release damage-associated molecular patterns (DAMPs) that promote dendritic cell (DC) maturation and T cell activation; when combined with immune checkpoint therapy, it can reduce the expression of PD-1, reverse hypoxia, and enhance efficacy; improving CAR-T therapy requires increasing its mitochondrial activity and plasticity, enhancing stemness and durability; targeting mitochondrial DNA can induce its oxidative release, activate the cGAS-STING pathway, and promote immune cell infiltration. These strategies contribute to enhancing anti-tumor immunity (**Table 1**).

**Table 1:** Features of mitochondria-targeted drugs on cancer immunotherapy.

No.	Parent compound	Mitochondrial target strategy	Mechanism of mitochondria-targeted drugs in tumor immunotherapy	Ref.
1	1G3-Cu and Toy	GCT NPs	Induces ICD, promotes DC maturation, and increases tumor-infiltrating cytotoxic T lymphocytes.	(253)
2	aCD24, CEL and shMFN1	P-aCD24/CEL ^+^ P/shMFN1	Activation of tumor cell phagocytosis improves macrophage-based immunotherapy.	(292)
3	ApSF	MSN	Induces ICD of tumor cells, promotes maturation of DCs and increases the number of infiltrating immune cells. Macrophages polarize from the M2 phenotype to the M1 phenotype, reducing the percentage of immunosuppressive Tregs.	(293)
4	Apt-LPR	cationic liposome	Generation of ROS to activate suppressive immune cells	(294)
5	Atorvastatin	CS-HAP@ATO NPs	Generates ROS and releases oxidized mitochondrial DNA (OX-mitoDNA). Activates inflammatory vesicles and enhances anti-tumor immune response.	(295)
6	Atovaquone		Inhibition of Foxp3 T cell differentiation and/or survival and promotion of Teff cell IFNγ Reduction of granulocyte MDSCs and regulatory T cells in the TME. Increase in tumor-infiltrating CD4^+^ T cells. Increases OXPHOS activity and aerobic glycolysis in activated CD8^+^ T. Inhibits OXPHOS and glycolysis in G-MDSC. Mitigates hypoxia and synergizes with ICB antibody against PD-L1 Tumor hypoxia normalization to enhance the efficacy of anti-PD-1 therapy.	(296) (237) (238) (271) (274)
7	Barium Titanate (BTO)	BTO/BP-HA	Inhibition of mitochondrial respiration promotes apoptosis in tumor cells and induces ICD, triggering an immune response.	(297)
8	BH3 analog		Enhancing NK-based immunotherapy	(298)
9	BQR	liposome	Promotes the release of mitochondrial DNA into the cytoplasm, activates the cGAS-STING pathway, and increases cross-presentation of antigens by phagocytosis of DCs and macrophages. Initiates CD8^+^ T cell infiltration into the TME.	(250)
10	Calcium phosphate	Ca@GOx	Induces mitochondrial Ca2^+^ overload and generates large amounts of ROS, induces cellular pyroptosis and promotes tumor infiltration of CD8^+^ T cells.	(242)
11	CaZCH	CaZCH NPs	Shifting TAM polarization toward the M1 phenotype induces ICD with M1, promotes DC maturation and activates CD8^+^ T cell-dependent systemic antitumor immunity.	(299)
12	Cinnamaldehyde	MON-CA-TPP@HA	Excess ROS activate oxidative stress, induces apoptosis and ICD, promotes DC maturation and CD8^+^ T cell activation, and regulates the M1/M2 macrophage ratio.	(300)
13	Ce6	BioPEGDMA	Enhances activation of CD3^+^/CD4^+^, CD3^+^/CD8^+^ T lymphocytes and DCs in tumor tissues and lymph glands.	(301)
14	cEMSY	Lipid nanoparticles	DNA leakage stimulates the cGAS-STING pathway	(302)
15	Co	TPP@CoTCPP	Activation of the cGAS-STING pathway induces an immune pro-inflammatory response effectively triggering an anti-tumor T cell response.	(303)
16	Comp. 4	TPP	Oxidative stress stimulates ICD response and triggers systemic anti-tumor immunity.	(304)
17	Cu (II)	PCD@CM	Induces significant immune surveillance, triggering ICD to promote cytotoxic T-lymphocyte infiltration and aPD-L1-mediated immune checkpoint blockade.	(305)
18	Cu (II) and TI	TPP	Downregulates PD-L1 and promotes intra-tumoral infiltration and activation of cytotoxic T lymphocytes.	(306)
19	CuET	TPP	Triggers immunogenic death, induces M1 polarization of macrophages, promotes antigen processing and presentation in cancer cells, and activates immune responses of CD8^+^ T cells and NK cells.	(256)
20	DCA	OPDEA-PDCA	Induces mitochondrial oxidative stress; produces immunogenic pyroptosis and prolongs T cell activation. Leads to lower lactate levels and regulates DC phenotype. Increases the number of IFN γ-producing CD8^+^ T cells and NK cells.	(218) (229) (307)
21	Decitabine	MDSC membrane vesicles	Induces mitochondrial damage and enhances ICD-mediated antitumor immunity. Reduces infiltration of MDSCs and M2 macrophages, increases proportion of CD4^+^, CD8^+^ T cells and CD103^+^ DCs	(308)
22	DZ@A7	MOF	Induces activation of the cGAS-STING pathway, promotes DC maturation and infiltration of cytotoxic T lymphocytes.	(289)
23	EGCG	IR780/Ce@EGCG/APT	Induces DC maturation, promotes cytotoxic T-lymphocyte infiltration, improves the immunosuppressive microenvironment, activates the systemic immune system, and generates long-term immune memory.	(247)
24	FDC and IR780	nanoparticle	Causes ICD, promotes DC maturation and increases the number of infiltrating immune cells. Polarizes M2 macrophages to the M1 phenotype and depletes MDSCs.	(257)
25	FFa		Generates mtROS, oxidizes mtDNA and promotes its leakage into the cytoplasm, resulting in the secretion of cGAS-STING-dependent IFN-I. Improves tumor antigen uptake, DC maturation, and CD8^+^ T cell cross-initiation.	(249)
26	FEPT	PEG2000-TPP	Phototherapy-induces hyperthermia or ROS, triggers the release of immunogenic intracellular substrates from dying tumor cells, which promotes the activation of antitumor immunity.	(246)
27	F-pY-T	Self-assembled nanoparticles	Induces ROS production and ICD, promotes DC maturation and intra-tumoral infiltration of tumor-specific T cells.	(309)
28	HMME and PTX	Liposome	Weakens hypoxic microenvironment, increases ROS levels and ICD.	(310)
29	Hydroxyurea (HU)	TPP	Inhibits MDSC and suppressor neutrophils and stimulates T cell responses	(235)
30	I3A	TPP nanocells	Induces ICD and activates adaptive immunity.	(311)
31	Indocyanine green (ICG)	SLNP/ICG@M	Activates proliferation of CD4^+^ T cells and CD8^+^ T cells.	(243)
32	Ir(iii) complexes	Nanoparticles	Oxidative stress production leads to disruption of the mitochondrial respiratory chain, which disrupts mitochondrial OXPHOS and glycolytic metabolism, and triggers cell death through combined ICD and ferritin phagocytosis	(234)
33	IR700DX-6T	TSPO-PDT	Induces ICD and activates dendritic and CD8^+^ T cells Generates ROS and induces cellular pyroptosis. Combined with a PD-1 blocker, triggers a potent anti-tumor immune response.	(312) (244)
34	IR780	PEG-PCL-IR780-TPZ NPs	This exacerbates the hypoxic microenvironment of the tumor, triggering ICD, accelerating DC maturation, and subsequently activating toxic T lymphocytes. Initiates ICD, DC maturation and synergistic T cell initiation	(313) (314)
35	LID	Liposomes	Induces oxidation of tumor mitochondrial DNA, translocates to APCs, activates cGAS-STING signaling, and triggers potent anti-tumor T cell immunity.	(291)
36	Lonidamine	TPP	Mitochondrial autophagic flux blockade induces and enhances pyroptosis, which promotes the release of immune-activating factors and the maturation of DCs.	(315)
37	Metformin		mtROS production stimulates IFN γ-dependent reprogramming in CD8^+^TILs. Reduces tumor-infiltrating Treg (TiTreg) Inhibits G-MDSCs Activates the Hippo signaling pathway to regulate PD-L1 Increases CD8^+^ T cell infiltration and survival in hypoxic tumor regions T cell metabolic reprogramming Combination with PD-1 blockers improves intra-tumoral T cell function and tumor clearance.	(316) (231) (317) (271) (239) (318)
38	Mi-2		Triggers ICD-associated immune activation and enhances CD8^+^ T-cell toxicity.	(319)
39	MiBaMc	TPP	Induces ICD, promotes DC maturation, and triggers T cell-mediated immune responses.	(320)
40	Mitochondria R		Regulates glycolysis and mitochondrial metabolism	(321)
41	Mito-CI	TPP	Inhibits the differentiation, viability, and suppressive function of bone marrow-derived MDSC and increases the proliferation-independent activation of T cells.	(237)
42	MNP	TPP	Leads to immunogenic death and activates immune responses in macrophages.	(130)
43	MNP-RGD-TPP	TPP	M1 polarizes and promotes DC maturation, and awakens cytotoxic T lymphocytes.	(322)
44	mPEI/M1mt		Elevates ROS accelerates the phosphorylation of NF-κB p65, MAPK p38, and JNK, which promotes M1 macrophage polarization, stimulates CD8^+^ and CD4^+^ T cell-dependent immune responses, and enhances the therapeutic effect of anti-PD-L1 treatment.	(323)
45	MTO and aPD-L1	RMP@Cap	Induces tumor cell pyroptosis and therefore triggers the release of mitochondrial DNA, enhances STING activation, and reduces inhibition of cytotoxic T cells.	(324)
46	Orlistat and anti-CD36	OB@D-pMOF/CaP-AC, DDS	Reprograms lipid metabolism and improves immune responses.	(325)
47	Om and As	Magnetic metal-organic framework	Modulation of mitochondrial function of CAFs and TILs to increase the level and activity of TILs.	(213)
48	P780 and KET	Nanoplatforms	Disrupts mitochondrial integrity and enhances ROS accumulation, triggering ICD.	(245)
49	PES		Induces DC activation	(326)
50	pheophorbide A and PXTK	mCAuNCs@HA	Induction of ICD activates CD4^+^, CD8^+^ T cells and NK cells	(327)
51	primary homing receptor p32	AKRGARSTA	Regulates tumor macrophages	(328)
52	PS TPAQ-Py-PF_6_ and PTX	versatile bionic nanoplatform	Induces ICD, the ability to initiate the cGAS-STING pathway promotes DC maturation and recruitment.	(329)
53	R162 and IR780	Liposome MLip RIR NPs	Causes severe iron death of tumor cells through accumulation of lipid peroxides. Triggers ICD, activates anti-tumor immunity, and suppresses primary and distant tumors with the help of immune checkpoint blockade.	(252)
54	R837	TPP, nMOF	Induces ICD, promotes DC activation	(255)
55	Raddeanin A (RA)		Promotes DC maturation and CD8^+^ T cell activation for tumor control	(330)
56	Resiquimod	nanocarrier	Targets TAMs for M1 phenotypic polarization. Triggers tumor ICD, DC maturation, TAM polarization and cytotoxic T lymphocyte infiltration.	(331)
57	Resveratrol		Induces CTLs and LAK cells, and produces cytokines IFN-γ, IL-2, TNF-α, and IL-12.	(332)
58	shMFN1 and DOX	MIX-NPs	Repolarizes TAMs from M2 to M1 phenotype. Triggers ICD, DCs, and promotes infiltration and activates CD8^+^ T cells. Suppresses MDSC and Tregs to further remodel ITM.	(333)
59	Silver	Ag@CuS-TPP@HA	Generates ROS that triggers ICD, leading to the massive release of TAA and DAMPs, improving the tumor immunosuppressive microenvironment and augmenting immune checkpoint blockade therapies.	(276)
60	siRNA	MMPA	Enhances ROS production, induces mitochondrial damage and mtDNA leakage into tumor tissue	(175)
61	SMAC-P, DOX	liposome	Induces robust ICD, promotes DC maturation and stimulates T-cell proliferation and activation, transforming the ITM into an immune response environment.	(254)
62	SMIP004-7		Enhances CD4^+^ and CD8^+^ T cell-mediated immune surveillance	(334)
63	SN-38	SN-38-TTCF@O2 NPs	Conducts ICD, promotes the recruitment and activation of cytotoxic T lymphocytes, and enhances the efficacy of anti-PD-1 antibody.	(335)
64	Tamoxifen	nanoparticle	Reversal of tumor hypoxia and inhibition of PD-L1 protein expression enhances the efficacy of photodynamic immunotherapy through enhanced T-cell infiltration. Effectively reduces PD-L1 and TGF-β expression in tumors by enhancing T-cell infiltration.	(255) (273)
65	TDV	TPP-HA-TDV	Enhances the release of ICD markers and subsequently induces immune responses	(336)
66	TLND	TPP	Triggers tumor ICD, induces DC maturation, promotes cytotoxic T cell infiltration, and modulates the TME.	(337)
67	TSPO	dendritic polymer	Stimulates anti-tumor immune signaling. Specifically targets mitochondria within TAM.	(338)
68	TT		Induces ICD, activates immune cell infiltration.	(339)
69	Zn-LDH		Promotes a pro-inflammatory network consisting of M1 tumor-associated macrophages, cytotoxic T cells, and NK cells. Activates the cGas-STING signaling pathway, induces ICD and induces antigen-specific cytotoxic T lymphocytes.	(340)
70	ZnPc	T-ZnPc-NPs	Leads to significant DC maturation and stimulates T cells to form cytotoxic CD8^+^ T cells.	(341)
71	Zoledronic acid (NZ)	nanoparticle	Increases diversity of anti-tumor infiltrating cells (Vγ9Vδ2 T lymphocytes, CD8^+^ T lymphocytes, NK cells)	(342)

## 5 Conclusion

In conclusion, mitochondria play a crucial role in tumor immunity. Abnormalities in mitochondria, such as genomic mutations, and autophagy can regulate cancer progression by regulating cellular metabolism and ROS production and may further confer competitive advantage to cancer cells. Under mitochondrial stress, mtDNA is released into the cytoplasm or extracellular fluid where it can be recognized by PRRs cGAS, TLR9, and NLRP3. Among them, the cGAS-STING signaling pathway plays a dual role in tumor immunity: it can suppress anti-tumor immunity to promote cancer progression, or enhance tumor antigen presentation to exert an anti-tumor effect. TLR9 promotes tumor growth and increases chemoresistance by recognizing the specific domain of mtDNA, whereas activated NLRP3 triggers downstream signaling cascades and assembles inflammasomes via potassium ion efflux.

Due to the elevated mitochondrial membrane potential (δψm) in tumor cells, DLCs including TPP and its derivatives, F16, Rhodamine and DQA can selectively accumulate in mitochondria and deliver loaded drugs. Specifically, TPP and its derivatives enhance the efficacy of chemotherapy, PDT, radiotherapy, and PTT; F16-targeted mitochondrial matrix induces mitochondrial permeability transition; Rhodamine stains mitochondria and enhances tumor suppression, and DQA disrupts mitochondrial function to induce cancer cell death. In terms of peptide-targeting sequences, MPPs consisting of 4–16 cationic and hydrophobic residues, can localize to mitochondria, promote apoptosis in cancer cells, and inhibit apoptosis in cancer cells, and shows low toxicity and good biocompatibility. SS peptides, which typically contain 4 positively charged amino acids, specifically target mitochondrial cardiolipin to reduce mitochondrial ROS production, inhibit MPTP turn-on, and protect mitochondrial structure and function.

These mitochondria-targeting strategies can also enhance tumor immunity through multiple mechanisms: targeting metabolic pathways (e.g., inhibiting key glycolytic enzymes such as GLUT1 and HK2, or modulating OXPHOS to reverse the Warburg effect; targeting mtROS to trigger ICD; targeting autophagy; combining immune checkpoint therapy to downregulate PD-1 expression; improving CAR-T cell therapy; and targeting mtDNA to induce their oxidative release and activate the cGAS-STING pathway, thereby promoting the infiltration of anti-tumor immune cells). Collectively, these strategies contribute to enhancing anti-tumor immunity.

Although mitochondria-targeting strategies have shown significant research potential and application prospects in the field of tumor immunity, providing an innovative direction for anti-tumor therapy, the development of anti-tumor drugs is still in the early stage, however, there are still several key challenges and limitations clinical application. On the one hand, the vast majority of studies on mitochondrial targeting are still in the preclinical stage, mainly focusing on *in vitro* cell experiments and *in vivo* animal model validation. The relative paucity of human clinical research data, especially the lack of support from multicentre, large sample phase III trials, makes it difficult to fully validate the effectiveness and applicability of these strategies in clinical cancer treatment. Further studies in the future include the following aspects: 1 How to regulate the activity and biological function of mitochondria in the activation of different immune cell subsets? 2 How does the energy balance transfer to immune cells? How to promote the repair of mitochondrial dysfunction and metabolic deficiency in immune cells while inhibiting tumor cell metabolism? Effective means for evaluating the safety of mitochondria-targeting drugs has not been established, and the potential risk of toxic side effects needs to be focused on. For example, some targeted molecules may have nonspecific effects on the mitochondria of normal cells, interfere with energy metabolism and cellular homeostasis in normal tissues, or induce immune-related adverse reactions. The above issues need to be addressed through in-depth mechanistic studies, dosage form optimization, and future clinical translation studies to promote the safe and effective application of mitochondria-targeted drugs in clinical anti-tumor therapy.

## Glossary

ATG: Autophagy-related genes
ACI: adoptive cellular immunotherapy
AML: acute myeloid leukemia
AMP: adenosine monophosphate
AMPK: AMP-activated protein kinase
ANT: adenine nucleotide translocase
ANXA1: annexin 1
APCs: antigen-presenting cells
ARG: arginase
As: astragaloside IV
ATM: ataxia telangiectasia mutated
ATP: adenosine triphosphate
BNIP3: BCL2 Interacting Protein 3
BNIP3L: BCL2 Interacting Protein 3 Like
BPD-MA: benzoporphyrin derivative monoacid ring A
BTO: Barium Titanate
CAR-T cell: Chimeric antigen receptor T cell
CD8+ T: cytotoxic T-cell
CDT: Chemodynamic therapy
CEBPβ: CCAAT Enhancer Binding Protein Beta
CGAS: Cyclic GMP-AMP Synthase
CRC: Colorectal cancer
CRT: calreticulin
CSF1: colony stimulating factor 1
CTSS: Cathepsin S
CuPpIX: Cu-modified protoporphyrin
CXCL1: C-X-C Motif Chemokine Ligand 1
Cytc: cytochrome c
DAMPs: damage-associated molecular patterns
DC: dendritic cells
DLC: delocalized lipophilic cation
DOX: doxorubicin
DQA: dequaliniumchloride
ECM: extracellular matrix
EGF: epidermalgrowthfactor
EPR: enhanced permeability and retention
ER: endoplasmic reticulum
FABP5: Fatty acid binding protein 5
FAO: Fatty acid oxidation
FFA: free fatty acids
Foxp3: Forkhead box P3
FUNDC1: FUN14 Domain Containing 1
G-CSF: Granulocyte-Colony Stimulating Factor
GLUT1: glucose transporter 1
GM-CSF: granulocyte macrophage-colony stimulating factor
GZMB: Granzyme B
HF: Hinokiflavone
HIF-1α: hypoxia-inducible factor-1α
HK2: hexokinase 2
HMGB1: high-mobility group box 1
HSP: heat shock protein
ICD: immunogenic cell death
ICG: Indocyanine green
IDO1: indoleamine2,3-dioxygenase1
IFN-I: type I interferon
IL: interleukin
ITM: immunosuppressive tumor microenvironment
iTreg: induced Treg
KRAS: KRAS Proto-Oncogene, GTPase
LAG-3: lymphocyte activation gene 3
LC3B: Microtubule-associated protein 1 light chain 3B
LDH-A: lactate dehydrogenase
LND: lonidamine
LPNPs: Lipid-polymer nanoparticles
MCHMs: macrophage-cancer hybrid membranes
MCJ: methylation-controlled J protein
MCT: monocarboxylate transporters
MDSC: myeloid-derived suppressor cell
Met: Metformin
MHC: major histocompatibility complex
MHC-Ⅰ: Major Histocompatibility Complex, Class I
MIF: migration inhibitory factor
Mito-: TPP-modified PEGylated mitochondrial-targeted ATO
Mito-CI: mitochondria-targeted complex I inhibitors
Mito-HU: Mitochondria-targeted hydroxyurea
Mito-LND: Mitochondria-targeted mito-LND
Mito-Q: Mitoquinone
MMP-9: matrixmetalloprotein 9
MMPA: membrane-permeable peptide amphiphiles
MOFs: Metal-organic frameworks
MOMP: membrane permeabilization
MPP: Mitochondria-penetrating peptide
mPTP: mitochondrial permeability transition pore
mtDNA: mitochondrial DNA
mTORC1: rapamycin complex 1
mtROS: mitochondrial ROS
MWCNTs: Multi-walled carbon nanotubes
NADPH: nicotinamide adenine dinucleotide phosphate
NEFL: Neurofilament Light Chain
NIR: near-infrared
NK: natural killer
NO: nitric oxide
NOX: NADPH-oxidase
NPs: nanoparticles
NZ: Zoledronic acid
O2·-: superoxide
OCT: organic cation transporters
Om: oxymatrine
OX-mitoDNA: oxidized mitochondrial DNA
OXPHOS: oxidative phosphorylation
PACSIN1: Protein Kinase C And Casein Kinase Substrate In Neurons 1
parkin: E3 ubiquitin-protein ligase parkin
PBA: Phenylboronic acid
PD-1: programmed cell death protein 1
PDHC: pyruvate dehydrogenase complex
PD-L1: programmed cell death ligand 1
PDT: Photodynamic therapy
PEG: polyethylene glycol
PEP: phosphoenolpyruvate
PET: phosphorescence energy transfer
PGC1α: peroxide-activated receptor 1α
PHDs: prolyl hydroxylases
PINK1: PTEN-induced putative kinase 1
PK: Pyruvate kinase
PKM2: pyruvate kinase-M2 splicing isoform
PLAC8: Placenta Associated 8
PRRs: pattern recognition receptors
pS6: phosphorylated S6
PTA: photothermal agents
PTT: Photothermal therapy
PTX: Paclitaxel
RA: Raddeanin A
RGS1: Regulator Of G Protein Signaling 1
RhodOA: Rhodamine B conjugated oleanolic acid derivatives
RNS: reactive nitrogen
ROS: reactive oxygen species
SDT: sonodynamic therapy
siRNA: small interfering RNA
SiR-PXZ: silicon rhodamine-based photosensitizer
SREBP: sterol regulatory element-binding protein
SS: Szeto-Schiller
STING: Stimulator Of Interferon Response CGAMP Interactor
TAA: tumor-associated antigens
TAM: tumor-associated macrophages
TAMs: tumor-associated macrophages
TCPP: tumor-targeted cell membrane penetrating peptides
TCR: T cell receptor
TDC: Targeted dendritic curcumin
TExh: T-cell exhaustion
TGF-: transforming growth factor beta
TILs: tumor-infiltrating lymphocytes
Ti-Treg: tumor-infiltrating Treg
TLR: toll-like receptors
TME: tumor microenvironment
TPP+: triphenylphosphine
TPPLs: DSPE-PEG-TPP polymer liposomes
Treg: regulatory T-cells
TSA: tumor-specific antigens
ULK1: UNC-51-like kinase 1
UPS: undifferentiated pleomorphic sarcoma
ZnPc: zinc phthalocyanine
ZVI-NPs: Zero-valent-iron nanoparticle
α-TOS: α-tocopheryl succinate
ΔΨm: mitochondrial membrane potential
